# Development and validation of protein biomarkers of health in grizzly bears

**DOI:** 10.1093/conphys/coaa056

**Published:** 2020-06-24

**Authors:** Abbey E Wilson, Sarah A Michaud, Angela M Jackson, Gordon Stenhouse, Nicholas C Coops, David M Janz

**Affiliations:** 1 Department of Veterinary Biomedical Sciences, University of Saskatchewan, 44 Campus Drive, Saskatoon, Saskatchewan S7N 5B3, Canada; 2 The University of Victoria Genome BC Proteomics Centre, 4464 Markham St #3101, Victoria, British Columbia V8Z 7X8, Canada; 3 Foothills Research Institute, Grizzly Bear Program, 1176 Switzer Drive, Hinton, Alberta T7V 1V3, Canada; 4 Department of Forest Resource Management, University of British Columbia, 2424 Main Mall, Vancouver, British Columbia V6T 1Z4, Canada

**Keywords:** Health, physiology, proteomics, *Ursus arctos*, wildlife monitoring

## Abstract

Large carnivores play critical roles in the maintenance and function of natural ecosystems; however, the populations of many of these species are in decline across the globe. Therefore, there is an urgent need to develop novel techniques that can be used as sensitive conservation tools to detect new threats to the health of individual animals well in advance of population-level effects. Our study aimed to determine the expression of proteins related to energetics, reproduction and stress in the skin of grizzly bears (*Ursus arctos*) using a liquid chromatography and multiple reaction monitoring mass spectrometry assay. We hypothesized that a suite of target proteins could be measured using this technique and that the expression of these proteins would be associated with biological (sex, age, sample location on body) and environmental (geographic area, season, sample year) variables. Small skin biopsies were collected from free-ranging grizzly bears in Alberta, Canada, from 2013 to 2019 (n = 136 samples from 111 individuals). Over 700 proteins were detected in the skin of grizzly bears, 19 of which were chosen as targets because of their established roles in physiological function. Generalized linear mixed model analysis was used for each target protein. Results indicate that sample year influenced the majority of proteins, suggesting that physiological changes may be driven in part by responses to changes in the environment. Season influenced the expression of proteins related to energetics, reproduction and stress, all of which were lower during fall compared to early spring. The expression of proteins related to energetics and stress varied by geographic area, while the majority of proteins that were affected by biological attributes (age class, sex and age class by sex interaction) were related to reproduction and stress. This study provides a novel method by which scientists and managers can further assess and monitor physiological function in wildlife.

## Introduction

Evaluation of biodiversity across the globe has revealed a significant decline in species abundance and the rate of such losses has continued to rise (Butchart *et al.*, 2010; Johnson *et al.*, 2017). The impact of anthropogenic resource use has been reported as a major driver of terrestrial vertebrate population decline, with potential cascading effects on ecosystem function (Dirzo *et al.*, 2014). Within terrestrial vertebrates, large carnivores play crucial roles in the maintenance and function of natural ecosystems, yet this group continues to experience population decline and range contraction across the globe (Ripple *et al.*, 2014b). In particular, grizzly bears (*Ursus arctos*) have been shown to provide top-down effects in terrestrial communities by influencing ungulate density, vegetation structure and avian populations (Bergee *et al*., 2001; Ripple *et al.*, 2014a). However, these habitats are often subject to anthropogenic resource use, including forestry, oil and gas exploration, mining, agriculture and recreational use, as is the case for grizzly bear habitat in west-central Alberta, Canada (Berland *et al.*, 2008; Proctor *et al.*, 2012). There has been a substantial effort to determine conservation strategies and manage recovery for this population (Berland *et al.*, 2008; Nielsen *et al.*, 2009; Boulanger and Stenhouse, 2014), as this group was listed as ‘threatened’ by the province in 2010 (Festa-Bianchet, 2010; Clark and Slocombe, 2011). Novel approaches to understanding biodiversity loss, such as the combination of occupancy modelling and indicators of physiological stress have proven useful (Semper-Pascual *et al.*, 2019); however, there is a lack of knowledge regarding specific biomarkers indicative of health in mammalian species. Biomarkers that are produced during specific physiological states may be used to detect compromised health in individuals and provide early warnings of decreasing individual and population performance in threatened wildlife (Clark *et al.*, 2001; Wikelski and Cooke, 2006).

Protein biomarkers found in blood, urine, skin and other matrices can be used to predict physiological and reproductive health (Barron *et al.*, 2015; Burke, 2016; Thomas *et al.*, 2016; Voiculescu *et al.*, 2016). For example, the relationship between prenatal stress, glucocorticoid concentrations and increased risk of disorders in adulthood has been extensively studied in human and animal models (Harris and Seckl, 2011). Specifically, environmental stress, undernutrition and exposure to excess stress-related hormones have been shown to lead to increased risk of metabolic, cardiovascular and neuropsychiatric disorders in human and animal model (rat and sheep) offspring (Seckl and Meaney, 2006; Meaney *et al.*, 2007). Affected offspring of model species (humans, rats, sheep) have demonstrated impaired physiological function in response to stress during adulthood and these effects have been shown to be transferred to one to two future generations (Seckl and Meaney, 2006; Mastorci *et al.*, 2009). Similar trends have been demonstrated in wild species, with mounting evidence to support that stress influences health, reproduction and disease susceptibility in individuals (Acevedo-Whitehouse and Duffus, 2009; Schultner *et al.*, 2013; Hing *et al.*, 2016).

The application of 21st century protein technologies used in human medicine to free-ranging wildlife species may address the need for the integration of wildlife health into conservation management. Such technologies include liquid chromatography and multiple reaction monitoring mass spectrometry (LC-MRM/MS) assays, which are capable of accurate quantitation of numerous proteins in complex systems within a single experiment (Vidova and Spacil, 2017). Proteins are functional molecules generated by the information expressed in the genome, and the expression of proteins is vital to cellular and biological function, thus providing a relevant characterization of a biological system (Cox and Mann, 2007). Approaches such as transcriptomics may not provide the most accurate biological response to stimuli, as mRNA levels are more transient and do not always correlate with protein expression, likely because regulatory processes occurring after mRNA is transcribed play a significant role in controlling protein abundances (Gygi *et al.*, 1999; Cutler, 2003; Vogel and Marcotte, 2012). LC-MRM/MS assays have been developed to detect and quantify specific target proteins for hypothesis-driven experiments. These assays provide higher reproducibility, sensitivity and selectivity compared to untargeted or global (‘shotgun’) proteomic approaches (Vidova and Spacil, 2017). An untargeted proteomic approach was recently used to determine pregnancy biomarkers in the faeces of captive cheetahs (*Acinonyx jubatus*) (Koester *et al.*, 2017). However, gathering similar samples that provide physiological information from free-ranging species often requires physical restraint and/or chemical immobilization of individuals, which in itself can result in an elevated stress response (Munerato *et al.*, 2010; Casas-Díaz *et al.*, 2012; Cattet *et al.*, 2014). Therefore, we suggest developing a technique to identify and measure biomarkers of health in skin, which can potentially be collected remotely (Karesh *et al.*, 1987; Pagano *et al.*, 2014).

Skin is a highly innervated organ that is exposed to a wide range of stressors throughout an individual’s lifetime. Skin and its appendages can act as both a target and source of key stress responses, making it a model organ for assessing the neuro-endocrine-immune response to stress (reviewed by Arck *et al.*, 2006). Skin is thought to have an independent and functional neuroendocrine system that expresses proteins, mediators and receptors similar to the hypothalamic-pituitary-adrenal (HPA) axis (Slominski *et al.*, 2000; Ito, 2005; Szöllősi *et al.*, 2017). This allows skin to respond to environmental stressors and maintain cutaneous homeostasis (Slominski *et al.*, 2000, 2018). The skin system also plays a role in the management of global homeostasis by communicating with the brain about changes in the epidermal environment and initiating responses from coordinating systems (Slominski *et al.*, 2012). There has been a significant effort to characterize the functional skin proteome with the aim of understanding disease and developing successful therapeutic strategies in humans (Huang *et al.*, 2005). In animal systems, the proteomic profiling of skin has been used to identify proteins related to physiological function (Geng *et al.*, 2015), responses to the environment (George *et al.*, 2010) and chemical communication (Leroy *et al.*, 2006). More recently, potential biomarkers of health and welfare have been identified in the skin proteome of fish (Sanahuja and Ibarz, 2015). Our laboratory developed an antibody-based protein microarray to identify and measure stress-associated proteins in the skin of grizzly bears (Carlson *et al.*, 2016); however, potential biomarkers of grizzly bear health determined by LC-MRM/MS have not been explored.

This study aimed to determine protein expression in skin of grizzly bears using LC-MRM/MS and conduct an initial evaluation of potential biomarkers of physiological function. We hypothesized that proteins related to energetics, reproduction and stress could be determined using this technique and that protein expression would be associated with biological (sex, age, sample location on body) and environmental (geographic area, season, sample year) variables. To test this hypothesis, the first objective of this study was to identify proteins that were detectable in the skin of grizzly bears, as skin has been shown to respond to environmental stimuli and play a role in maintaining global homeostasis. Given that specific proteins can be biomarkers of physiological function and reproductive status, the second objective of this study was to determine the effects of biological (sex, age, sample location on body) and environmental (geographic area, season, sample year) variables on 19 target proteins related to energetics, reproduction and stress. To our knowledge, this is the first study to identify and measure proteins related to these functional categories in grizzly bears and relate the changes in protein expression to biological and environmental parameters.

## Material and methods

### Animals

**Figure 1:**
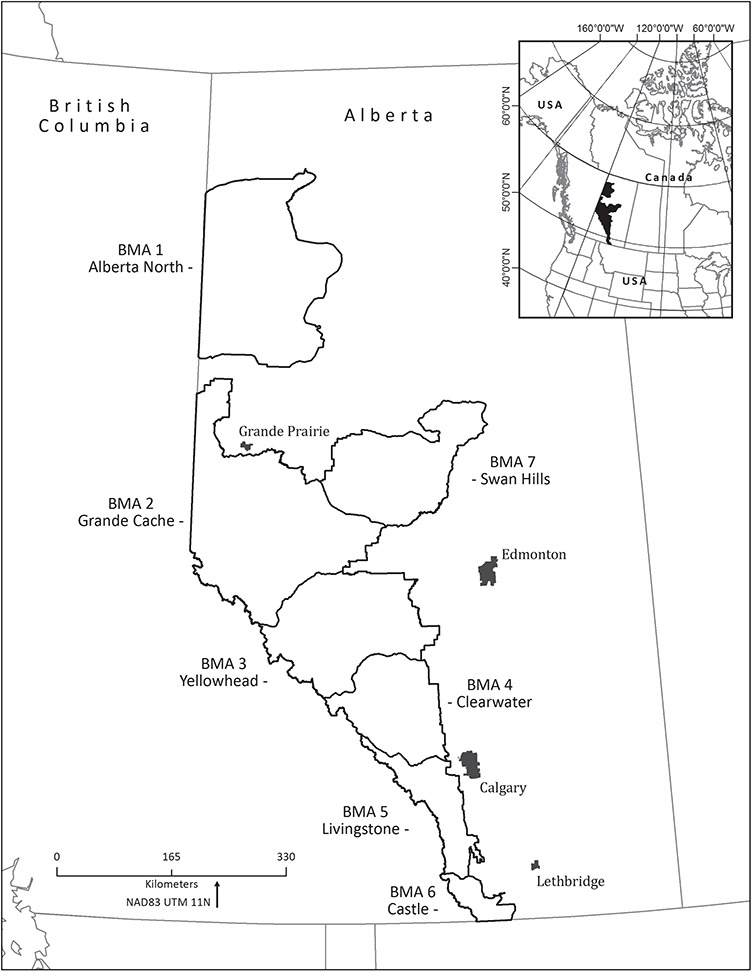
Study area location in Alberta, Canada. Skin samples were collected from grizzly bears (*U. arctos*) captured across six BMAs (all except Swan Hills) from 2013 to 2019.

We conducted this study with free-ranging grizzly bears (*U. arctos*) in Alberta, Canada. Grizzly bears were officially listed as threatened by the province in 2010 (Festa-Bianchet, 2010; Clark and Slocombe, 2011), with populations bounded by the major east-west transportation corridors (Proctor *et al.*, 2012). Seven provincial bear management areas (BMAs) were created between these highways in order to manage population recovery. Samples were collected from individuals captured in six of the BMAs: Grande Cache, Yellowhead, Clearwater, Livingstone, Castle and Alberta North (Fig. 1). Grizzly bears were captured by the Foothills Research Institute (fRI Research) Grizzly Bear Program (Hinton, Alberta, Canada). fRI Research has been monitoring and conducting research on this free-ranging grizzly bear population for the past 21 years. The program lead and primary capture team members have been consistent over the lifetime of this program. All personnel were trained in the standardized protocol for the capture, handling and sample collection of grizzly bears according to the fRI Research Protocol for the Capture, Handling and Sampling of Grizzly Bears. Furthermore, sample collection was standardized and did not change between years. In brief, individuals were captured by either remote drug delivery from a helicopter (aerial darting) or the ground (ground darting), or culvert trap. All bears were anesthetized by remote drug delivery (Pneu-Dart Inc., Williamsport, Pennsylvania, and Dan-Inject, Kolding, Denmark) using a combination of medetomidine and Telazol (Fort Dodge Laboratories, Inc., Fort Dodge, Iowa) and the effects of medetomidine were reversed by administering atipamezole (Antisedan; Novartis Animal Health Canada Inc., Mississauga, Ontario, Canada). A series of health parameters (pulse and respiratory rates, rectal temperature and haemoglobin oxygen saturation) were recorded at the start of handling and every 15 minutes throughout the handling period. For further details on capture and handling procedures see Cattet *et al.* (2008). Body condition index (BCI), weight and straight-line length of individuals was recorded when possible (see Supplementary Table S1.1). See Cattet *et al.* (2002) for information on how BCI was calculated. BCI, weight and straight-line length values for all bears fell within three standard deviations of the mean for each season, sex and age category (e.g. hypophagia, female, subadult). All captures were authorized by Alberta Environment and Parks and Parks Canada and research and collection permits were obtained on an annual basis. All capture and handling procedures were based on the Canadian Council of Animal Care and the American Society of Mammologists guidelines and were approved annually by the University of Saskatchewan’s Committee on Animal Care and Supply and by the Alberta Environment and Parks Animal Care Committee (Animal use Protocol Number 20010016).

### Sample collection and preparation

A single skin sample was either collected from the outside of the upper thigh (area above the knee on the hind leg) and/or from the external ear surface (pinna) of a grizzly bear using a 4–6-mm biopsy punch (see Supplementary Fig. S1.1). Biopsy sites were shaved and cleaned with povidone-iodine solution prior to collection and the biopsy wound was covered with topical ointment (Carlson *et al.*, 2016). All skin samples were collected during April to October from 2013 to 2019. The majority of samples were collected from bears captured in a culvert trap (82), followed by aerial darting (20), ground darting (6) and immediately after a mortality (3). Samples were collected approximately 20 minutes post aerial darting and approximately 10 minutes after remote drug delivery from the ground or within a culvert trap. Skin samples were placed into a cryovial, labelled and immediately placed in a cooler on ice. If possible, dry ice was generated using a portable dry ice maker at the trap site and samples were frozen on dry ice within 20 minutes of collection. Otherwise, samples were frozen at −20°C within 1–4 h of collection and all samples were kept frozen for 0–7 days until being transferred to long-term storage at −80°C. In order to mitigate protein degradation, it is recommended to snap freeze tissue samples when possible or keep cool and/or frozen until samples can be transferred to long term storage at −80°C. Biospecimens can be stored at −80°C for several years (7–17 years) with no changes in tissue integrity (reviewed by Shabihkhani *et al.*, 2014). Furthermore, the activity of specific proteins has also been shown to be unaffected by storage in liquid nitrogen or at temperatures of −70°C or −20°C (McLeay *et al.*, 1992). Sample quality was assessed for all samples by quantifying the total protein yield, as a poor yield would suggest tissue degradation (Shabihkhani *et al.*, 2014).

All hair, fat and muscle were removed from the skin under liquid nitrogen using pre-chilled forceps and scalpels and the weight of the remaining skin sample was recorded. Samples were ground to a fine powder under liquid nitrogen using pre-chilled mortars and pestles and stored at −80°C until processed further. The subsequent protein extraction and digestion methods as well as the LC-MRM/MS methods were completed at The University of Victoria Genome BC Proteomics Centre (Victoria, British Columbia, Canada).

### Discovery analysis

Discovery analyses were completed with three samples collected from one female and two male grizzly bears in order to determine which putative proteins (and corresponding peptides) were detectable in skin. Discovery (shotgun) proteomics is used to simultaneously identify hundreds of proteins in a biological matrix and often provides the basis for target protein selection (Liu *et al.*, 2004; Michalski *et al.*, 2011; Vidova and Spacil, 2017). Proteins were digested into peptides by trypsin using a traditional urea-based in-solution digestion method as well as a commercial kit using the in-StageTip (iST; Preomics, München, Germany) method (described in further detail below) (Vandermarliere *et al.*, 2013; Pasing *et al.*, 2017; Sielaff *et al.*, 2017). The peptide mixtures were separated by on-line reverse phase chromatography using a Thermo Scientific EASY-nLC II system with a Magic C18-AQ reversed-phase pre-column (100 μm I.D., 2.5 cm length, 5 μm, 100 Å) and an in-house prepared Magic C-18AQ reversed phase nano-analytical column (75 μm I.D., 15 cm length, 5 μm, 100 Å; Michrom BioResources Inc, Auburn, CA, USA) at a flow rate of 300 nl/min. Solvents were as follows: A, 2% acetonitrile, 0.1% formic acid; and B, 90% acetonitrile, 0.1% formic acid. Following column equilibration, samples were separated by a 100 min gradient (0 min: 5%B; 90 min: 30%B; 2 min: 100%B; hold 8 min: 100%B).

The chromatography system was coupled on-line with an LTQ Orbitrap Velos-Pro mass spectrometer (Thermo Fisher Scientific, Bremen, Germany) equipped with a Nanospray Flex source (Thermo Fisher Scientific) set at a voltage of 2.5 kV and a capillary temperature of 250°C. The survey MS1 scan m/z range was from 400–2000 in profile mode with a resolution of 60 000 full width at half maximum (FWHM) at 400 m/z with an automatic gain control target of 1e6 and one microscan with a maximum inject time of 500 ms. The 10 most intense ions charge state 2–4 exceeding 25 000 counts were selected for collision-induced dissociation (CID) ion trap MS/MS fragmentation (ITMS scans 2–11) with detection in centroid mode.

### Data analysis and protein identification from discovery analysis

To identify proteins indicative of health that were detectable in grizzly bear skin, the initial raw files from the discovery analysis were created by XCalibur 2.2 (Thermo Scientific) software and analyzed with Proteome Discoverer 1.4.0.228 software suite (Thermo Scientific). Parameters for the spectrum selection to generate peak lists of the CID spectra included a signal to noise ratio cut-off of 1.5, a total intensity threshold of 0, a minimum peak count of 1 and a precursor mass range of 350–5000 Da. The peak lists were submitted to an in-house Mascot 2.4.1 server Universal Protein Resource (UniProt) Knowledge Base database 20 161 219 (71 594 789 sequences; 24 046 658 667 residues) and compared against sequences identified in species of Caniformia. UniProt provides a comprehensive and accurately annotated protein sequence database that is accessible to the scientific community (Bateman, 2019). Caniformia or Canoidea (‘dog-like’) is a suborder within the order Carnivora, that includes Canidae (dogs, wolves, foxes), Ursidae (bears), Pinnipedia (seals, sea lions, fur seals, walrus) and musteloidea (red pandas, skunks, badgers, raccoons, weasels). In brief, the peak search parameters included a precursor tolerance of 8 ppm, a MS/MS tolerance of 0.6 Da, allowance of 1 missed cleavage of the trypsin enzyme, a fixed modification of carbamidomethylation (C) and variable modifications of deamination (N,Q) and oxidation (M).

Analyses of the Proteome Discover result files were performed with the Scaffold Q + S software package (Proteome Software, Inc, Portland, OR) to identify target proteins that represented energetics, reproduction and stress. Because this study aimed to test biologically plausible hypotheses related to the identification of potential health biomarkers, we narrowed the total list of proteins to focus on specific target proteins indicative of health because of their established relation to energetics, reproduction and stress. We focused our search on proteins that matched known sequences in species of Ursidae, while removing proteins that matched sequences found in the giant panda (*Ailuropoda melanoleuca*), as grizzly bears are more closely related to the polar bear (*Ursus maritimus*) and black bear (*Ursus americanus*). Next, we selected proteins that were detected in 2/3 samples (except Transthyretin, which was only detected in one sample) and had a protein identification probability >65% in each sex (male and female). An extensive literature review was completed to determine the biological function of each protein. Proteins were then grouped by biological function and the list was further revised to include those that were potential biomarkers of health status related to energetics, reproduction and stress. This resulted in a total of 19 proteins selected as targets for the LC-MRM/MS assay (Table 1). The majority of target proteins contained three proteotypic peptides (a peptide sequence that is found in only a single known protein and, therefore represents that protein); however, three proteins (adiponectin, ceruloplasmin, kininogen-1) contained peptide sequences that were also found in other isoforms of the same protein. All peptide sequences were identified in the UniProt database (Hinxton Cambridge, UK; Bateman, 2019) and matched sequences identified in *U. maritimus*, the polar bear.

**Table 1:** Target proteins identified and quantified in grizzly bear skin

Category	Protein (*U. maritimus*)	Accession number	Biological function	Biomarker	Reference
Energetics	Adiponectin	A0A384D8N0_URSMA	Regulates glucose	Metabolic disease	(Esmaili *et al.*, 2018)
	Clusterin	A0A384D8F3_URSMA	Transports cholesterol and clears cellular debris	Degenerative diseases and tumorigenesis	(Ishaq *et al.*, 2017)
	Apolipoprotein B-100	A0A384CVD9_URSMA	Transports lipids	Cardiovascular disease	(Bali and Utaal, 2019)
	Alpha-1-acid glycoprotein	A0A384DLK6_URSMA	Carries exogenous and endogenous substances in bloodstream	Inflammation and liver disease	(Bteich, 2019)
	Transthyretin	A0A384C416_URSMA	Transports thyroid hormones and retinol	Protein-calorie malnutrition	(Tóthová and Nagy, 2017)
	Vitamin D-binding protein	A0A384CAI2_URSMA	Transports vitamin D metabolites	Liver and renal disease; pregnancy	(Bikle and Schwartz, 2019)
Reproduction	Ceruloplasmin	A0A384DEX8_URSMA	Carries copper	Pregnancy	(Linder, 2016)
	Fetuin-B	A0A384D9G1_URSMA	Inhibits calcium phosphate precipitation	Female fertility	(Dietzel *et al.*, 2016)
	Complement C3	A0A384DSD2_URSMA	Activates complement system	Impaired immune response	(Ricklin *et al.*, 2016)
	Afamin	A0A384CAW5_URSMA	Transports vitamin E metabolites	Pregnancy; metabolic disease	(Dieplinger and Dieplinger, 2015)
	Prostaglandin (PG) F synthase 1	A0A384D9Z8_URSMA	Reduces PGD_2_ to PGF2 for muscle contraction and parturition	Pregnancy and parturition	(Helliwell *et al.*, 2004)
	Serpin B5 (maspin)	A0A384D874_URSMA	Suppresses tumors	Pregnancy-associated disorders	(Khalkhali-Ellis, 2006)
Stress	78 kDa glucose-regulated protein (GRP78/BIP)	A0A384CR94_URSMA	Regulates protein folding	Stress; pathogenesis	(Ibrahim *et al.*, 2019)
	Endoplasmin (HSP90B1)	A0A384DAW8_URSMA	Maintains protein homeostasis	Stress; pathogenesis	(Hoter *et al.*, 2018)
	Superoxide dismutase (SOD)	A0A384CZ25_URSMA	Antioxidant	Inflammatory and infectious disease	(Carillon *et al.*, 2013)
	Corticosteroid-binding globulin (CBG)	A0A384D132_URSMA	Transports cortisol	Acute and chronic inflammation	(Meyer *et al.*, 2016)
	Alpha-2-macroglobulin	A0A384CLK6_URSMA	Inhibits proteases	Inflammatory and liver disease	(Rehman *et al.*, 2013)
	Kininogen-1	A0A384D8Y8_URSMA	Regulates blood coagulation	Inflammatory disease	(Göbel *et al.*, 2018)
	Annexin	A0A384C464_URSMA	Reduces inflammation of tissues	Inflammatory disease	(Bruschi *et al.*, 2018)

### Development and validation of target protein and peptide panel

A panel of 19 proteins were quantitated by peptide-based analysis using LC-MRM/MS. Tryptic peptides were selected to serve as molecular surrogates for the 19 target proteins according to a series of peptide selection rules (e.g. sequence uniqueness, devoid of oxidizable residues, (see Kuzyk *et al.*, 2013 for detailed criteria) and previous detectability in bear species samples).

To compensate for matrix-induced suppression or variability in LC-MS performance, stable isotope-labelled standard (SIS) peptides were generated by adding an amino acid labelled with the stable isotopes, ^13^C and ^15^N, to each endogenous peptide. These labelled peptide analogues were used as internal standards, as SIS peptides differ in mass compared to their unlabelled counterparts, but not in other chemical or physical properties (Kettenbach *et al.*, 2011). Light (unlabelled) peptides were used to construct an external calibration curve for quantification of the endogenous peptide in tissue samples. All peptides (light and SIS) were synthesized via Fmoc (9-fluorenylmethoxycarbonyl) chemistry, purified [through reversed phase-high performance liquid chromatography (RP-HPLC) with subsequent assessment by matrix-assisted laser desorption/ionization time-of-flight mass spectrometry (MALDI-TOF-MS)] and characterized via amino acid analysis and capillary zone electrophoresis (Percy *et al.*, 2014; Michaud *et al.*, 2018). Additional details describing the synthesis of peptides are provided elsewhere (Percy *et al.*, 2013). The peptides were validated for their use in LC-MRM/MS experiments following The National Cancer Institute’s Clinical Proteomic Tumor Analysis Consortium (CPTAC; https://assays.cancer.gov/) guidelines for assay development (Michaud *et al.*, 2018).

### Protein extraction and optimization for LC-MRM/MS assay

In order to optimize protein extraction, we tested 10 separate extraction and digestion methods on sections (11.4–21.0 mg) of skin collected from a bulk sample (see Supplementary Fig. S1.2). In addition to traditional in-solution methods [urea, deoxycholate (DOC) and sodium dodecyl sulfate (SDS)], two commercially available kit-based approaches were used: iST and S-Trap (Protifi, Huntington NY, USA) (see Pasing *et al.*, 2017 for example workflows). Total protein content for each method was determined by the Bradford assay (BioRad Protein Assay, Hercules, CA, USA). In the Bradford assay, protein is bound to Coomassie Brilliant Blue G-250, causing a shift in absorbance that can be measured using a spectrophotometer (Bradford, 1976). Out of all sample preparation methods, 4.5 M urea, 9 M urea and 5% SDS produced similar total protein concentrations (approximately 180 μg/mg tissue; see Supplementary Table S1.2). Ultimately, the workflow using 9 M urea was chosen because of ease of handling and fewer negative downstream effects on the mass spectrometer. Furthermore, all endogenous peptides were adequately released during digestion, allowing their detection on the mass spectrometer when using the 9 M urea workflow.

The homogenized samples were rehydrated in 4.5 M urea and 300 mM tris(hydroxymethyl)aminomethane (Tris) to a pH of 8.0 and centrifuged at 13300 rcf for 10 min to remove any insoluble material. Proteins from the soluble fractions were precipitated overnight at −20°C in five volumes of acetone. The samples were centrifuged at 80000rcf for 5 min at 4°C before decanting the acetone and rehydrating the protein pellets in 9 M urea and 300 mM Tris to a pH of 8.0. Protein concentration was determined for each sample as well as a pooled sample using the Bradford Assay.

### Protein digestion

Sample order was randomized and a volume corresponding to 50 μg of skin protein from each sample and the pool was transferred to a 96-well microplate (Axygen Scientific, Inc., Union City, CA, USA). The disulfide bonds were reduced for 30 min at room temperature using dithiothreitol (DTT, 20 mM final) and cysteine residues alkylated for 30 min at room temperature in the dark using iodoacetamide (IAA, 40 mM final). The urea concentration was diluted to 1 M with 100 mM Tris prior to proteolysis by the addition of N-tosyl-L-phenylalanine chloromethyl ketone (TPCK)-treated trypsin (5 μL at 1 mg/mL; Worthington Biochemical Corporation, Lakewood, NJ, USA) at a 20:1 substrate:enzyme ratio (Vandermarliere *et al.*, 2013). After an 18-hr incubation at 37°C, proteolysis was quenched with 10% formic acid. The SIS peptides were then spiked into the digested individual and pooled samples, the standard curve samples and the curve quality control (QC) samples. The standard curve was prepared using a mix of light peptides that was spiked into a bovine serum albumin tryptic digest spanning a concentration range of 1 to 1000× the assay’s lower limit of quantitation (LLOQ) over eight dilutions. The curve QC samples were prepared from the same light peptide mix and spiked in bovine serum albumin digest at 4×, 40× and 400× the LLOQ for each peptide. Samples were subsequently concentrated by solid phase extraction (SPE; Oasis HLB, 2 mg sorbent; Waters Corporation, Milford, MA, USA). After SPE, the concentrated eluate was frozen, lyophilized to dryness (approximately 4 hours) and rehydrated in 0.1% formic acid (final concentration: 1 μg/μL digest).

### LC-MRM/MS assay for targeted analysis

Injections (20 μL) of the grizzly bear skin tryptic digests were separated with a Zorbax Eclipse Plus RP-UHPLC column (2.1 × 150 mm, 1.8 μm particle diameter; Agilent Technologies, Santa Clara, CA, USA) that was contained within a 1290 Infinity LC system (Agilent Technologies, Santa Clara, CA, USA). Peptide separations were achieved at 0.4 mL∕min over a 60 min run, via a multi-step LC gradient (2–80% mobile phase B; mobile phase compositions: A was 0.1% formic acid in water while B was 0.1% formic acid in acetonitrile). The column temperature was maintained at 50°C. A post-gradient equilibration of 4 min was used after each sample analysis.

The LC system was interfaced to a triple quadrupole mass spectrometer (Agilent 6495; Agilent Technologies, Santa Clara, CA, USA) via a standard-flow electrospray ionization (ESI) source, operated in positive ion mode. The general MRM acquisition parameters employed were as follows: 3.5 kV capillary voltage, 300 V nozzle voltage, 11 L∕min sheath gas flow at a temperature of 250°C, 15 L∕min drying gas flow at a temperature of 150°C, 30 psi nebulizer gas pressure, 380 V fragmentor voltage, 5 V cell accelerator potential and unit mass resolution in the first and third quadrupole mass analyzers. The high energy dynode multiplier was set to −20 kV for improved ion detection efficiency and signal-to-noise ratios. Peptides were empirically optimized by analysis of the purified SIS peptides using Skyline-daily Quantitative Analysis software (version 19.1.1.248, MacCoss Laboratory, University of Washington, Seattle, WA, USA). The resulting peptide specific acquisition parameters were employed for optimal peptide ionization/fragmentation and scheduled MRM. In the quantitative analysis, the targets (five transitions/peptide; one quantifier and four qualifiers) were monitored over 900 ms cycles with 1 min detection windows. For a step-by-step guide on the development of multiple reaction monitoring mass spectrometry assays and a general review on mass spectrometry-based quantitative proteomics, see Vidova and Spacil, 2017.

### Quantitative analysis of target proteins

In order to quantitate the 19 target proteins, the MRM data was first visualized and examined with Skyline-daily Quantitative Analysis software. This involved peak inspection to ensure accurate selection, integration and uniformity (in terms of peak shape and retention time) of the SIS and light peptide forms. The pooled sample was analyzed for both sample preparation and MS analysis consistency across the 96-well plate. After defining a small number of criteria (i.e. 1/x^2^ regression weighting and <20% deviation in the curve QC’s level’s accuracy) the standard curve was used to calculate the peptide concentration in fmol/injection of skin protein in samples through linear regression using the light to SIS peak area ratios (Percy *et al.*, 2014; Michaud *et al.*, 2018). The calculated concentrations were further normalized by the amount of total protein injected (μg of sample) into the instrument. The concentration of each protein is represented by one proteotypic peptide; therefore, a series of steps were taken to determine which peptide would be used for quantitation. First, 10 (out of 47) peptides were removed from analysis because >25% of values were below the LLOQ; however, each protein still had at least one proteotypic peptide for statistical analysis. Second, since peptides are released during digestion with different efficiencies, proteins were represented by the peptide with the greatest average concentration across all samples, thus representing the most accurate concentration of the protein. Furthermore, a total of 6 (out of 136) samples were removed from further analysis because >25% of values were below the LLOQ, resulting in a total of 130 samples with 37 corresponding peptides (1–3 peptides per protein) for statistical analysis. All values below the LLOQ were replaced with half the lowest quantifiable value in the data set for each peptide (Knott *et al.*, 2011).

To determine the influence of specific attributes on 19 target proteins related to energetics, reproduction and stress, we used generalized linear mixed model (GLMM; Zuur *et al.*, 2013) analysis to evaluate the effects of biology (sex, age class and their interaction), geographic area (BMA), sample year (2013–2019), season (hypophagia, early hyperphagia and late hyperphagia) and sample location on the body [biopsy from thigh, ear and other (shoulder and unknown location)] on the mean expression for each of the target proteins. The relationships between responses (mean protein expression in fmol of protein/μg of sample) and independent variables were modelled using a γ distribution and log link (Carlson *et al.*, 2016). Samples were collected from 111 individuals, 17 of which had more than one sample collected (130 samples total); therefore, individual was included in the models as a random effect to account for the variation between different bears. Multiple samples were collected from the same individual in different years or from different locations on the body within the same year. Slope graphs for individual bears that show the relationship between repeated samples across years (skin samples collected from the outer thigh) are provided in Supplementary Fig. S1.3. Sex was divided into three categories: male, female and female with cubs, as grizzly bear home range size, movement and denning chronology differ among these groups, all of which may impact physiological function in individuals (Graham and Stenhouse, 2014; Carlson *et al.*, 2016). Age class was divided into adults (≥5 years old), subadults (<5 years old) and yearlings (<2 years old). The interaction effect of sex and age class (adult female, adult female with cubs, subadult female, yearling female, adult male and subadult male) was also tested. We used seasons based on grizzly bear feeding habits (Nielsen *et al.*, 2004a; Carlson *et al.*, 2016), where (i) hypophagia is the period from den emergence (typically in April) to 14 June; (ii) early hyperphagia is from 15 June to 7 August; and (iii) late hyperphagia is from 8 August to den entry, which is typically in November.

We used Akaike’s information criterion corrected for small sample size (AIC_c_) to determine the best model, represented by the lowest AIC_c_ for all model combinations (Semper-Pascual *et al.*, 2019) and calculated the difference in AIC_c_ values between all additive model combinations and the best model (Burnham and Anderson, 2002). For models that demonstrated a change in AIC_c_ ≤ 2 (Burnham and Anderson, 2002), we calculated the average of parameter estimates with 95% confidence intervals (CIs) across competing models (Burnham and Anderson, 2002; Arnold, 2010; Hill *et al.*, 2019). A covariate was considered to have a significant effect if the 95% CI excluded zero. Both marginal R^2^ and conditional R^2^ were calculated to evaluate model fit. Reference categories for each parameter (sex: male; age: adult; season: hypophagia; BMA: Yellowhead; sample year: 2013; and type: thigh) were used for all analyses. We completed model development in R 3.5.3 statistical software (R Development Core Team, 2013) using the ‘glmer’ function in package ‘lme4’ (Bates *et al.*, 2014; Carlson *et al.*, 2016). All protein concentrations are shown as mean fmol of protein per μg of sample.

## Results

**Figure 2:**
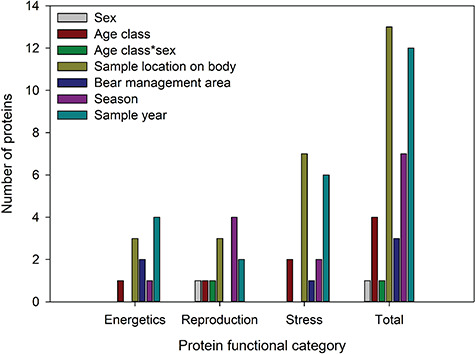
Number of proteins in each functional category significantly (*P* < 0.05) influenced by biological (sex, age, sample location on body) and environmental [bear management area (geographic area), season, sample year] variables. Proteins were measured in skin samples collected from grizzly bears (*U. arctos*) in Alberta, Canada from 2013 to 2019.

An initial untargeted analysis of three skin samples revealed amino acid sequences from an average of 782 ± 132 proteins with homologous sequences found in species belonging to the sub-order Caniformia. Structural proteins, enzymes related to glucose metabolism and several protease inhibitor proteins were detected in skin. Models were developed for each target protein (related to energetics, stress, reproduction) to determine which biological and environmental variables were predictors of mean protein expression (see Supplementary Tables S1.3–S1.5). We found that sample location on the body and sample year influenced the greatest number of proteins, followed by season, age class, BMA and sex and sex by age class (Fig. 2).

### Proteins related to energetics

Sample year had the greatest influence on proteins related to energetics (4/6 proteins), followed by sample location on the body (3/6 proteins), BMA (2/6 proteins), season (1/6 proteins) and age class (1/6 proteins). The significant (*P* < 0.05) associations between predictor variables and the expression of proteins related to energetics are listed in Table 2. The mean expression of adiponectin was greater during 2015 and 2016 compared to 2013, while the mean expression of clusterin, transthyretin and vitamin D-binding protein was less in 2018 and 2019 compared to 2013. In comparison to samples collected from the thigh, the mean expression of alpha-1-acid glycoprotein and vitamin D-binding protein decreased when collected from the ear. Conversely, the mean expression of alpha-1-acid glycoprotein and transthyretin was greater when collected from other areas of the body (shoulder or unknown). The mean expression of clusterin and transthyretin were greater in the Clearwater BMA compared to the Yellowhead BMA. The mean expression of Vitamin D-binding protein was reduced during late hyperphagia compared to hypophagia. Lastly, the mean expression of alpha-1-acid glycoprotein was higher in sub adults compared to adults. The expression of apolipoprotein B-100 was not associated with any biological or environmental variables. Furthermore, sex and the sex by age class interaction did not have an effect on any proteins within the energetics group.

**Table 2:** Generalized linear mixed model results of the influence of biological and environmental factors on the mean expression of proteins related to energetics in grizzly bear skin (n = 6 proteins; n = 111 individuals)

	Adiponectin			Clusterin			Apolipoprotein			Alpha-1-acid glycoprotein			Transthyretin			Vitamin D		
	β	95% CI		β	95% CI		β	95% CI		β	95% CI		β	95% CI		β	95% CI	
**Intercept**	**1.66**	**1.27**	**2.04**	**2.09**	**1.56**	**2.61**	−0.13	−1.03	0.76	**2.75**	**2.50**	**3.01**	**3.26**	**2.84**	**3.68**	**3.45**	**3.10**	**3.80**
**Sex**																		
Female							0.05	−0.26	0.35	0.14	−0.27	0.56						
FC							−0.19	−0.83	0.44	0.02	−0.29	0.33						
**Age**																		
Subadult	0.07	−0.19	0.33	0.20	−0.20	0.60	0.70	−0.03	1.43	**0.56**	**0.18**	**0.95**						
Yearling	0.09	−0.56	0.73	0.04	−0.83	0.90	0.94	−0.79	2.67	0.75	−0.64	2.14						
**Sample location on body**																		
Ear	−0.01	−0.23	0.21				−0.12	−0.52	0.28	**−0.29**	**−0.55**	**−0.04**	−0.28	−0.56	0.00	**−0.33**	**−0.56**	**−0.11**
Other	0.39	−0.18	0.97				0.25	−0.53	1.03	**0.52**	**0.09**	**0.95**	**0.67**	**0.16**	**1.18**	0.02	−0.39	0.44
**BMA**																		
Alberta North				−0.16	−1.72	1.40	0.09	−1.92	2.11				−0.14	−1.66	1.38			
Castle				0.19	−0.85	1.23	0.80	−0.64	2.24				0.37	−0.65	1.39			
Clearwater				**1.28**	**0.58**	**1.98**	1.12	−0.16	2.39				**1.54**	**0.88**	**2.21**			
Grande Cache				−0.24	−0.62	0.15	−0.07	−0.54	0.40				0.06	−0.30	0.42			
Livingstone				−0.48	−1.38	0.41	−1.44	−3.13	0.26				−0.06	−0.89	0.77			
Unknown				−0.23	−1.94	1.48	−0.69	−2.86	1.49				−0.18	−1.84	1.47			
**Season**																		
Early hyperphagia				−0.09	−0.49	0.31	0.03	−0.27	0.33							−0.03	−0.47	0.40
Late hyperphagia				−0.17	−0.60	0.26	−0.07	−0.40	0.27							**−0.42**	**−0.69**	**−0.15**
**Sample year**																		
2014	0.22	−0.18	0.62	0.01	−0.41	0.43	0.88	−0.09	1.85				−0.02	−0.43	0.39	0.10	−0.27	0.47
2015	**0.79**	**0.35**	**1.22**	−0.06	−0.53	0.42	0.46	−0.27	1.20				0.13	−0.33	0.59	0.27	−0.13	0.67
2016	**0.50**	**0.04**	**0.96**	−0.05	−0.54	0.45	0.27	−0.42	0.96				−0.09	−0.57	0.39	0.10	−0.33	0.52
2017	0.25	−0.25	0.76	−0.08	−0.58	0.42	0.59	−0.25	1.42				−0.13	−0.64	0.38	0.07	−0.39	0.52
2018	0.34	−0.14	0.82	**−0.69**	**−1.25**	**−0.14**	−0.01	−0.74	0.72				**−0.66**	**−1.19**	**−0.12**	0.07	−0.36	0.50
2019	−0.40	−1.00	0.20	**−2.26**	**−3.12**	**−1.39**	−0.91	−2.20	0.39				**−2.42**	**−3.27**	**−1.57**	**−0.96**	**−1.54**	**−0.39**
**Marginal R** ^**2**^	0.19			0.29						0.13			0.36			0.26		
**Conditional R** ^**2**^	0.75			0.73						0.77			0.76			0.78		

Model averaged parameter estimates and confidence intervals (95% CI) for models with sample size corrected Akaike’s information criterion (ΔAICc) <2 are shown. All models include bear ID as a random effect. Bold numbers indicate the CI does not include zero. Reference categories for each parameter (sex: male; age: adult; season: hypophagia; BMA: Yellowhead; year: 2013; type: thigh) are not shown. The parameter sex*age class was not included in any of the top models. Abbreviations are as follows: FC: female with cubs; BMA: bear management area; Vitamin D: Vitamin D-binding protein.

### Proteins related to reproduction

The expression of proteins related to reproduction was most associated with season (4/6 proteins), followed by sample location on the body (3/6 proteins), sample year (2/6 proteins), sex by age class, age class and sex (1/6 proteins). Table 3 shows the significant (*P* < 0.05) associations between predictor variables and the expression of proteins related to reproduction. The mean expression of ceruloplasmin, fetuin-B, complement C3 and afamin decreased during late hyperphagia compared to hypophagia. The expression of fetuin-B, complement C3 and afamin was reduced when samples were collected from the ear rather than the thigh. The expression of fetuin-B and afamin was less in samples collected during 2019 compared to 2013. Subadult females demonstrated a greater mean expression of prostaglandin F synthase 1 compared to adult males, while samples collected from subadults showed a significant increase in the mean expression of fetuin-B compared to adults. Lastly, the mean expression of ceruloplasmin was less in samples collected from females with cubs compared to males. Serpin B5 was not associated with biological or environmental variables. Furthermore, BMA did not have a significant effect on any proteins within the reproduction category.

**Table 3:** Generalized linear mixed model results of the influence of biological and environmental factors on the mean expression of proteins related to reproduction in grizzly bear skin (n = 6 proteins; n = 111 individuals)

	Ceruloplasmin			Fetuin-B			Complement C3			Afamin			Prostaglandin F synthase 1			Serpin B5		
	β	95% CI		β	95% CI		β	95% CI		β	95% CI		β	95% CI		β	95% CI	
**Intercept**	**1.42**	**1.18**	**1.67**	**1.87**	**1.32**	**2.42**	**2.03**	**1.53**	**2.52**	**1.96**	**1.47**	**2.46**	**−0.58**	**−0.95**	**−0.20**	**2.22**	**1.98**	**2.46**
**Sex**																		
Female	0.01	−0.37	0.40	0.13	−0.33	0.58				0.00	−0.15	0.15				0.05	−0.20	0.31
FC	**−0.73**	**−1.22**	**−0.25**	−0.02	−0.37	0.32				−0.07	−0.43	0.29				−0.11	−0.52	0.30
**Age**																		
Subadult				**0.89**	**0.41**	**1.37**	**0.53**	**0.16**	**0.90**	0.16	−0.26	0.58	0.08	−0.36	0.52	0.04	−0.19	0.27
Yearling				0.41	−1.33	2.16	0.21	−1.26	1.69	0.24	−0.78	1.27	−0.21	−1.65	1.23	0.10	−0.57	0.78
Sex*Age Class																		
Adult female													−0.06	−0.74	0.62			
Adult FC													−0.37	−1.03	0.28			
Subadult female													**1.00**	**0.23**	**1.77**			
Yearling female													−0.15	−1.38	1.08			
Subadult male													0.05	−0.32	0.42			
**Sample location on body**																		
Ear	−0.32	−0.64	0.00	**−0.30**	**−0.56**	**−0.04**	**−0.51**	**−0.77**	**−0.25**	**−0.40**	**−0.65**	**−0.16**						
Other	0.54	−0.02	1.10	0.40	−0.08	0.88	0.06	−0.42	0.55	0.17	−0.29	0.62						
**BMA**																		
Alberta North										0.13	−1.03	1.29						
Castle										0.35	−0.76	1.45						
Clearwater										0.55	−0.78	1.89						
Grande Cache										0.01	−0.26	0.27						
Livingstone										−0.03	−0.70	0.65						
Unknown										0.12	−1.20	1.43						
**Season**																		
Early Hyperphagia	0.06	−0.52	0.63	−0.30	−0.90	0.30	0.02	−0.53	0.57	−0.23	−0.75	0.29	−0.09	−0.59	0.41	0.50	−0.15	1.15
Late Hyperphagia	**−0.43**	**−0.77**	**−0.08**	**−0.67**	**−1.07**	**−0.27**	**−0.48**	**−0.84**	**−0.12**	**−0.63**	**−1.00**	**−0.26**	−0.02	−0.23	0.20	0.33	−0.08	0.73
**Sample year**																		
2014				0.02	−0.46	0.50	**0.76**	**0.29**	**1.22**	0.24	−0.18	0.66						
2015				−0.16	−0.73	0.41	0.35	−0.19	0.89	0.25	−0.22	0.72						
2016				−0.03	−0.65	0.58	0.43	−0.14	0.99	0.19	−0.31	0.70						
2017				−0.30	−0.98	0.38	0.38	−0.24	0.99	0.10	−0.46	0.65						
2018				−0.21	−0.86	0.43	0.25	−0.32	0.82	−0.13	−0.84	0.57						
2019				**−1.68**	**−2.44**	**−0.93**	−0.48	−1.18	0.22	**−1.26**	**−2.35**	**−0.16**						
**Marginal R** ^**2**^	0.18			0.34			0.24			0.28			0.12					
**Conditional R** ^**2**^	0.74			0.88			0.83			0.80			0.78					

Model averaged parameter estimates and confidence intervals (95% CI) for models with sample size corrected Akaike’s information criterion (ΔAICc) <2 are shown. All models include bear ID as a random effect. Bold numbers indicate the CI does not include zero. Reference categories for each parameter (sex: male; age: adult; season: hypophagia; BMA: Yellowhead; year: 2013; type: thigh) are not shown. Abbreviations are as follows: FC: female with cubs; BMA: bear management area.

**Table 4:** Generalized linear mixed model results of the influence of biological and environmental factors on the mean expression of proteins related to stress in grizzly bear skin (n = 7 proteins; n = 111 individuals)

	GRP78/BIP			Alpha-2-macroglobulin			Annexin			CBG			Endoplasmin			Kininogen			SOD		
	β	95% CI		β	95% CI		β	95% CI		β	95% CI		β	95% CI		β	95% CI		β	95% CI	
**Intercept**	**1.91**	**1.42**	**2.40**	**1.84**	**1.58**	**2.11**	**3.13**	**2.73**	**3.53**	0.03	−0.51	0.57	**0.71**	**0.28**	**1.14**	**1.81**	**1.30**	**2.32**	**3.29**	**2.97**	**3.62**
**Sex**																					
Female	0.14	−0.33	0.61				0.12	−0.23	0.47	0.02	−0.18	0.23				0.07	−0.25	0.39			
FC	0.02	−0.26	0.29				−0.03	−0.31	0.25	−0.02	−0.23	0.19				0.01	−0.21	0.22			
**Age**																					
Subadult	0.48	−0.13	1.09	**0.49**	**0.13**	**0.86**				0.27	−0.31	0.86	**0.52**	**0.20**	**0.83**	0.30	−0.19	0.80			
Yearling	0.30	−1.01	1.61	0.07	−1.43	1.56				0.13	−1.19	1.44	0.66	−0.61	1.93	0.11	−1.00	1.22			
Sex*Age class																					
Adult Female	0.29	−0.44	1.01							−0.09	−0.60	0.43									
Adult FC	0.05	−0.29	0.40							−0.22	−0.78	0.34									
Subadult female	0.25	−0.54	1.03							0.58	−0.40	1.57									
Yearling female	0.15	−0.68	0.98							0.05	−0.90	1.00									
Subadult male	0.14	−0.42	0.70							0.08	−0.27	0.43									
**Sample location on body**																					
Ear	−0.16	−0.40	0.09	**−0.49**	**−0.85**	**−0.13**	−0.04	−0.31	0.22	**−0.33**	**−0.58**	**−0.08**	−0.04	−0.28	0.21	**−0.54**	**−0.85**	**−0.23**	−0.20	−0.48	0.08
Other	**0.73**	**0.29**	**1.17**	0.01	−0.57	0.60	**0.65**	**0.19**	**1.11**	0.04	−0.41	0.50	**0.60**	**0.18**	**1.03**	−0.13	−0.67	0.41	**0.51**	**0.03**	**0.99**
**BMA**																					
Alberta North																0.00	−1.78	1.78			
Castle																0.62	−0.55	1.78			
Clearwater																**1.49**	**0.73**	**2.24**			
Grande Cache																−0.06	−0.47	0.35			
Livingstone																−0.48	−1.49	0.53			
Unknown																−0.08	−2.01	1.84			
**Season**																					
Early hyperphagia	0.22	−0.30	0.74	0.16	−0.45	0.77	0.11	−0.29	0.52	**−0.73**	**−1.41**	**−0.06**									
Late hyperphagia	−0.23	−0.63	0.17	**−0.58**	**−0.94**	**−0.22**	−0.09	−0.36	0.19	**−0.72**	**−1.15**	**−0.28**									
**Sample year**																					
2014	0.09	−0.37	0.55				0.27	−0.16	0.70	**0.76**	**0.29**	**1.23**	**0.43**	**0.03**	**0.84**	0.03	−0.42	0.49	−0.23	−0.63	0.16
2015	**0.57**	**0.07**	**1.07**				**0.80**	**0.35**	**1.25**	**0.74**	**0.20**	**1.28**	**0.84**	**0.38**	**1.30**	0.22	−0.29	0.72	0.30	−0.10	0.70
2016	0.37	−0.19	0.94				**0.59**	**0.11**	**1.07**	0.58	−0.03	1.19	**0.71**	**0.20**	**1.21**	0.05	−0.48	0.59	0.25	−0.18	0.67
2017	0.14	−0.44	0.73				**0.52**	**0.02**	**1.01**	0.36	−0.28	0.99	0.51	−0.03	1.04	−0.34	−0.92	0.24	−0.15	−0.57	0.27
2018	0.34	−0.22	0.90				**0.55**	**0.05**	**1.06**	0.02	−0.60	0.65	**0.59**	**0.08**	**1.11**	**−0.98**	**−1.60**	**−0.36**	−0.04	−0.47	0.38
2019	**−0.81**	**−1.45**	**−0.18**				−0.04	−0.59	0.52	−0.68	−1.42	0.06	−0.27	−0.89	0.35	**−2.53**	**−3.50**	**−1.56**	**−0.81**	**−1.31**	**−0.31**
**Marginal R** ^**2**^	0.24			0.16			0.19			0.36			0.26			0.38			0.20		
**Conditional R** ^**2**^	0.80			0.75			0.78			0.85			0.81			0.76			0.79		

Model averaged parameter estimates and confidence intervals (95% CI) for models with sample size corrected Akaike’s information criterion (ΔAICc) <2 are shown. All models include bear ID as a random effect. Bold numbers indicate the CI does not include zero. Reference categories for each parameter (sex: male; age: adult; season: hypophagia; BMA: Yellowhead; year: 2013; type: thigh) are not shown. Abbreviations are as follows: FC: female with cubs; BMA: bear management area; GRP78/BIP: 78 kDa glucose-regulated protein; CBG: corticosteroid-binding globulin; SOD: superoxide dismutase.

### Proteins related to stress

The majority of proteins related to stress were significantly (*P* < 0.05) influenced by sample location on the body (7/7 proteins) and sample year (6/7 proteins), followed by season and age class (2/7 proteins) and BMA (1/7 proteins). The significant (*P* < 0.05) associations between predictor variables and the expression of proteins related to stress are shown in Table 4. When samples were collected from another location on the body (shoulder or unknown), the mean expression of 78 kDa glucose-regulated protein (GRP78/BIP), annexin, endoplasmin and superoxide dismutase (SOD) increased compared to when samples were collected from the thigh. Conversely, when samples were collected from the ear, the mean expression of alpha-2-macroglobulin, corticosteroid-binding globulin (CBG) and kininogen was reduced compared to samples collected from the thigh. In 2014, the mean expression of CBG and endoplasmin was greater compared to 2013, while the mean expression of GRP78/BIP, annexin, CBG and endoplasmin was higher in 2015 compared to 2013. In 2016, the mean expression of annexin and endoplasmin was greater compared to 2013. The mean expression of annexin was also greater during 2017 compared to 2013. In 2018, the mean expression of annexin and endoplasmin increased compared to 2013; however, the mean expression of kininogen decreased compared to 2013. The mean expression of GRP78/BIP, kininogen and SOD was reduced in 2019 compared to 2013. The mean expression of alpha-2-macroglobulin and CBG decreased during late hyperphagia and the mean expression of CBG was also decreased during early hyperphagia compared to hypophagia. Similar to proteins in the energetics group, the expression of kininogen increased in samples collected from Clearwater compared to the Yellowhead BMA. Samples collected from subadults demonstrated an elevated mean expression of endoplasmin and alpha-2-macroglobulin compared to samples collected from adults. All stress-related proteins showed associations with biological or environmental variables; however, sex did not have an effect on any of the proteins in this category.

## Discussion

Monitoring the physiological function of free-ranging bears may provide important information regarding population health and help to identify factors that may be influencing energetics, reproduction and stress in individuals; however, methods to do this are lacking. While studies are limited, there have been attempts to identify protein biomarkers in animals including those indicative of stress in grizzly bears (Carlson *et al.*, 2016) and those indicative of pregnancy in domestic dogs (Kuribayashi *et al.*, 2003), cheetahs (Koester *et al.*, 2017), several wild canid species (Bauman *et al.*, 2008), black bears (Foresman and Daniel Jr, 1983) and polar bears (Curry *et al.*, 2012). Therefore, this study aimed to fill this gap by (i) identifying proteins that were detectable in the skin of grizzly bears; and (ii) determining initial relationships between biological (sex, age, sample location on the body) and environmental (geographic area, season, sample year) variables and the expression of proteins related to energetics, reproduction and stress. Sample location on the body and sample year influenced the greatest number of proteins overall (13/19 and 11/19 proteins, respectively). Other major findings from this study were that (i) season influenced the expression of proteins related to energetics, reproduction and stress, all of which were lower during late hyperphagia compared to hypophagia; (ii) the expression of proteins related to energetics and stress were greater in the Clearwater BMA compared to Yellowhead; (iii) the majority of proteins that were affected by biological attributes (age class, sex and age class by sex interaction) were related to reproduction and stress. To our knowledge, this is the first study to measure target proteins in grizzly bear skin related to energetics, reproduction and stress. This study provides the initial method development and broad associations with biological and environmental variables in order to potentially further assess physiological function in individuals and monitor species-at-risk that reside on changing landscapes.

### Anatomical sample location

In the current study, sample location on the body [biopsy from thigh, ear and other (shoulder and unknown location)] influenced the greatest number of proteins across all functional categories. Adiponectin, clusterin, ceruloplasmin and prostaglandin F synthase 1 were the only proteins not affected by sample location on the body. If samples are collected from multiple locations on the body, proteins that demonstrate consistent expression between sampling areas may be the most useful biomarkers. In all cases, when samples were collected from the ear, the mean protein expression was decreased compared to the thigh and when samples were collected from another location (shoulder or unknown), mean protein expression was increased. Our previous work reported that expression of 32 proteins did not differ among skin samples collected from the neck, forelimb, hindlimb and ear of grizzly bears; however, this was in a small subset of bears (n = 4) and a different analytical technique was used (Carlson *et al.*, 2016). Variability in protein expression between samples collected from the external ear (pinna) versus the outside of the upper thigh may be explained by differences in the anatomy and physiology of these two areas on the body. The external ear (pinna) is comprised of cartilage and covered by a thin layer of skin, while the upper thigh consists of fat and muscle that is enclosed by thick layers of skin (Akers and Denbow, 2013). The expression of proteins in skin could arise from either blood circulation or local synthesis in surrounding cells (Jonak *et al.*, 2006; Ono *et al.*, 2017); however, the relative contributions of each source are not known in the current study. Blood flow is more available in the upper thigh compared to the external ear, allowing for skin to have a higher and faster turnover rate; therefore, it is not surprising that protein concentrations would be greater in skin samples collected from the thigh. Samples collected from the shoulder or another unknown location likely came from an area of high fat and muscle, similar to the upper thigh. For these reasons, previous studies recommend using a biopsy punch on areas of the animal with high muscle mass, such as the rump, thigh or shoulder (Karesh *et al.*, 1987; Mijele *et al.*, 2016).

### Year and geographic area

Sample year influenced the expression of the majority of proteins in this study (three related to energetics, two related to reproduction, six related to stress). Proteins across all functional categories showed a general increasing pattern from 2014 to 2017 compared to 2013 and a decreasing pattern from 2018 to 2019 compared to 2013. In particular, proteins related to energetics and stress were elevated from 2014 to 2017 and decreased in 2018 and 2019, suggesting that factors related to environmental conditions and/or change over time such as snow cover (Berman *et al.*, 2019), natural disturbances (wildfire; Kearney *et al.*, 2019) and anthropogenic disturbance (Larsen *et al.*, 2019) could be influencing physiological function in grizzly bears. Increased seasonal food availability, such as the abundance and occurrence of berries and fruits, has been associated with more clearings and forest opening in anthropogenic disturbed areas (Nielsen *et al.*, 2016; Kearney *et al.*, 2019; Larsen *et al.*, 2019). Grizzly bears have been found to select these areas, which can result in an increase in human contact and reduced bear survival rates (McLellan and Hovey, 2001b; Nielsen *et al.*, 2004, 2004a, 2004b; Berland *et al.*, 2008). Increased food availability may also be contributing to a decreased stress response in recent years. Studies have reported hair cortisol concentrations in grizzly bears decreased after a year of high dietary salmon (Bryan *et al.*, 2013) and were also reduced with improved body condition (Macbeth *et al.*, 2012; Bourbonnais *et al.*, 2014).

The expression of proteins related to energetics (clusterin and transthyretin) and stress (kininogen) were significantly (*P* < 0.05) elevated in samples collected from the Clearwater BMA compared to Yellowhead. Within our study area, 4603 km^2^ of forest were disturbed by forest harvest, well sites, fires and road development from 2001–2011 (White *et al.*, 2014). However, grizzly bears residing in areas that have been impacted by human disturbance showed a reduction in hair cortisol concentrations (Bourbonnais *et al.*, 2013; Wilson *et al.*, 2020), which may be explained by changes in food availability and distribution in areas with anthropogenic disturbance (von der Ohe *et al.*, 2004; Wasser *et al.*, 2004; Bryan *et al.*, 2013). While this study demonstrates that sample year and geographic area influences protein expression in grizzly bears, we suggest further investigation into changes in the environment in those years and within each BMA that were not taken into account in this study, such as climatic variation, anthropogenic disturbance and changing distribution and abundance of bears.

### Sampling season

The expression of all proteins influenced by season demonstrated a decrease during late hyperphagia (fall) compared to hypophagia (spring), which may indicate that physiological function in grizzly bears could be explained by seasonal changes, such as food availability, mating behavior and the onset of hibernation activities (or a combination of these). Season influenced the expression of a single protein within the energetics group, vitamin D-binding protein, which is responsible for binding the principal vitamin D metabolites (Vitamin D_3_ and D_2_) and regulating the amount of bioavailable vitamin D (White and Cooke, 2000). Vitamin D plays an essential role in growth, reproduction and health by regulating calcium homeostasis in the body (Grant and Holick, 2005; Gernand *et al.*, 2016). Vitamin D can be obtained from the diet and/or sunlight exposure to the skin (Norman, 2008); therefore, we would expect vitamin D levels in grizzly bears to be lowest at the time of den emergence. However, levels of vitamin D_2_ were reported to be higher during winter in grizzly bears, while levels of vitamin D_3_ were higher in the summer (Vestergaard *et al.*, 2011). Furthermore, vitamin D obtained from the diet may be more important for bears, as their thick coat and skin may be decreasing cutaneous vitamin D production (Kenny *et al.*, 1998). Within our study area, grizzly bears have been reported to select for roots such as, sweet vetch (*Hedysarum*), and ungulate matter during hypophagia and at the beginning of early hyperphagia. During early hyperphagia the grizzly bear diet consists of green vegetation (horsetails, graminoids, forbs), while during the start of late hyperphagia, grizzly bears select for berries (*Shepherdia canadensis* and *Vaccinium parvifolium*) until fall, when bears would switch back to sweet vetch roots until den entry (Munro *et al.*, 2006). Grizzly bears living in coastal areas with access to marine resources likely receive vitamin D from salmon and other nutrient-rich foods; however, the mixed diet consumed by bears in our study area (interior bear populations) allows them to balance a high protein diet with carbohydrates in fruit, which may be an adaptive metabolic strategy (Rode and Robbins, 2000; Felicetti *et al.*, 2003; Coogan *et al.*, 2014). Therefore, we suggest the monitoring of vitamin D between and among populations with different feeding habits may provide insight on the metabolism and energetic characteristics of populations.

Reproductive proteins (ceruloplasmin, fetuin-B, complement C3, afamin) also decreased during late hyperphagia compared to hypophagia. These proteins are typically associated with the immune system and inflammation and have been found to increase during pregnancy in giant pandas and humans (ceruloplasmin; Derzsy *et al.*, 2010; Willis *et al.*, 2011), during successful fertilization in humans (fetuin-B; Floehr *et al.*, 2016) and during the early stages of pregnancy when individuals are developing preeclampsia (afamin; Köninger *et al.*, 2018). In our study area, grizzly bears typically mate from mid-May to the end of July, with a peak in mid-June (Stenhouse *et al*., 2005); however, this timeframe may be shifted due to changes in geographic photoperiod, metabolic state and social interaction (Steyaert *et al.*, 2012). Following a successful mating event, the fertilized ova will remain dormant for approximately 3 to 6 months until implantation occurs (Spady *et al.*, 2007). Therefore, if mating and subsequent fertilization occurs in May–June, then implantation would likely occur in November–December, making it difficult for managers and scientists to know if a female is pregnant before entering the den. In the current study, these reproductive proteins were decreased during the fall months overall; however, this may be because all sexes and ages were included in the study. In a previous study, concentrations of reproductive hormones were measured in the hair of adult grizzly bears only and were also found to fluctuate seasonally. Specifically, progesterone levels were reported to increase after breeding and into hibernation in breeding females, while testosterone and estradiol remained at low levels during post-breeding and hibernation (Cattet *et al.*, 2017). Therefore, we suggest further investigation of these tentative fertilization and pregnancy biomarkers by focusing on high and low reproducers with known mating and pregnancy status, a feat that is difficult to achieve with free-ranging animals. Seasonal changes in pregnancy biomarkers may be used to evaluate pregnancy status and the likelihood of a successful pregnancy.

The expression of proteins related to stress, alpha-2-macroglobulin and CBG, decreased during late hyperphagia compared to hypophagia. Alpha-2-macroglobulin is an integral part of the innate immunity system and inhibits enzymes that breakdown other important proteins (Rehman *et al.*, 2013). This protein has been shown to increase during hibernation in black bears, suggesting it has a protective role against metabolic depression (Sheikh *et al.*, 2003). Similarly, we found that the expression of alpha-2-macroglobulin was elevated during den emergence compared to the fall, which may be a result of high levels during hibernation. CBG is responsible for the transport of cortisol, which is released in response to increased stress (Meyer *et al.*, 2016) and may serve as a biomarker of chronic stress in grizzly bears (Chow *et al.*, 2010). CBG has been found to increase in fasting male polar bears compared to non-fasting males, suggesting that CBG may also play a role in the metabolic state by reducing free cortisol concentrations (Chow *et al.*, 2011). Grizzly bears also endure a period of fasting during hibernation and CBG expression was elevated upon den emergence, suggesting that levels were higher during a time of stressed metabolic state.

### Sex and age of individuals

The expression of alpha-1-acid glycoprotein, endoplasmin and alpha-2-macroglobulin was elevated in subadults compared to adults, which may be explained by the changing social interaction and dispersal of young grizzly bears. During this time, subadult bears leave the family unit and must establish their own home ranges while encountering dominant bears and other animals, searching for mates and locating food resources. Males tend to disperse farther than females (McLellan and Hovey, 2001a) and establish home ranges larger in size than females (Dahle *et al.*, 2006). However, because of the numerous overlapping home ranges, grizzly bears of both sexes are likely to experience interaction with other age classes and sexes. In our study area, interaction between subadults and adults of the same and opposite sex occur, especially interactions between adult and subadult females, which made up greater than 50% of the female-female associations (Stenhouse *et al*., 2005). Furthermore, subadult bears have been found closer to high-use roads and consequently, were subject to a higher encounter rate with humans and of being killed by humans (Mueller *et al.*, 2004; Graham *et al.*, 2010). The changing social interactions for subadult grizzly bears may affect individual health, represented by changes in the expression level of these energetic and stress-related proteins. While the expression of proteins related to stress was not influenced by sex in the current study, previous studies have reported differences in hair cortisol concentrations between male and female grizzly bears (Bourbonnais *et al.*, 2013).

The expression of ceruloplasmin, a protein that has been found to increase during pregnancy (Derzsy *et al.*, 2010; Willis *et al.*, 2011), was not influenced by the age class and sex interaction; however, regardless of age class, mean expression was lower in females with cubs compared to males. In Alaska, USA, females with cubs have been reported to avoid habitats with a high prevalence of adult males and select for areas with increased human presence, which is thought to mitigate the risk of infanticide (Rode *et al.*, 2006). Therefore, it is not surprising that proteins related to pregnancy would be lower in females that are not sexually active. The expression of prostaglandin F synthase 1 (PGF1) was higher in subadult females compared to adult males and fetuin-B was elevated in subadults compared to adults. The product of PGF1 (prostaglandin F_2α_) induces the degradation of the corpus luteum at the end of the luteal phase of the estrous cycle if pregnancy did not occur (Helliwell *et al.*, 2004) and has been used to monitor and influence the estrous cycle in domestic animals (Shirasuna *et al.*, 2012; Coffman and Pinto, 2016) and to predict pregnancy and parturition in giant pandas (Roberts *et al.*, 2018). In the current study, a subadult was defined as ≤5 years of age; however, this delineation is typically based on the age of primiparity and/or the age when a female produces cubs (Garshelis *et al.*, 2005). Other studies suggest that successful litter production can occur at 4 years of age (G. Stenhouse, personal communication, 2019; Schwartz *et al.*, 2003), suggesting that females would show signs of estrus in the previous mating season. The mean expression of PGF1 was highly variable among subadult females in the current study. Furthermore, swollen vulvas were observed during the capture of two subadult females in this study (ages 2 and 4), which may be indicative of mating events. Given the substantial role that prostaglandins and other reproductive-related proteins play in the estrous cycle and the high variability in our data, we suggest that increased expression of these proteins may be related to reaching sexual maturity. The age of sexual maturity is an important parameter to monitor when attempting to recover a population and can provide insight on the reproductive potential, status and social structure of a population (Schwartz *et al.*, 2003).

## Limitations and strengths of study

This study aimed to demonstrate an initial evaluation of potential biomarkers of physiological function in grizzly bears. However, a broader range of variables are needed to identify direct relationships between protein expressions, changes in physiological function and potential drivers of physiological function. While this study was limited by small sample sizes within some groups, unknown reproductive and pregnancy status of females and potential sample age and storage effects, this method was able to determine broad associations between protein expression and biological and environmental factors. This initial exploratory analysis will be followed up with a further study that statistically explores more detailed analyses of the data, including proteins expressed in the serum of females with known reproductive status. To move this technique forward, sample collection via remote biopsy darting may be useful for monitoring the health of individuals. Remote collection of samples eliminates the need to capture individuals, which can result in an elevated stress response (Munerato *et al.*, 2010; Casas-Díaz *et al.*, 2012; Cattet *et al.*, 2014). Although in the current study protein expression differed in ear samples, in practice, it is highly unlikely that a remote dart would target this tissue. Given the negative impacts capture has on health, remote biopsy darting of the same location (i.e. areas of high muscle mass, such as the thigh) may be useful for the practical application of this technique in the future.

The measurement of proteins in the current study can be applied to understanding and monitoring bear health and physiology in the context of both population recovery and landscape change. Furthermore, these novel techniques can be used as sensitive conservation tools to detect new threats to the health of individual animals well in advance of population-level effects by providing an early warning of population decline. We suggest that this method is well-suited for assessing the health of and monitoring wildlife, particularly those species-at-risk that reside on changing landscapes.

## Conclusions

This study provides a methodology for determining potential biomarkers of physiological function in wildlife. The current study used a liquid chromatography and multiple reaction monitoring mass spectrometry assay as a novel means to determine the expression of proteins related to energetics, reproduction and stress in grizzly bear skin. Results indicate that sample year influenced the majority of proteins, suggesting that physiological changes may be driven in part by responses to changes in the environment. Season influenced the expression of proteins related to energetics, reproduction and stress, all of which were lower during fall compared to early spring. The expression of proteins related to energetics and stress varied by geographic area, while the majority of proteins that were affected by biological attributes (age class, sex and age class by sex interaction) were related to reproduction and stress.

## Supplementary material

Supplementary material is available at *Conservation Physiology* online.

## Funding

This work was supported by the Grizzly-PAW project (Natural Sciences and Engineering Research Council of Canada (file: CRDPJ 486175–15; grantee: N.C. Coops, FRM, UBC), in collaboration with fRI Research and FRIAA, Alberta Newsprint Company, Canfor, Cenovus, Repsol, Seven Generations Energy, Shell Canada, TransCanada Pipelines, Teck Resources, West Fraser, Westmoreland Coal and Weyerhaeuser. More information can be found at http://paw.forestry.ubc.ca/.

## Supplementary Material

CONPHYS-2019-167_R2_Supplementary
