# Supplementary material for: Development and validation of protein biomarkers of health in grizzly bears
Source: Conserv Physiol. 2020 Jun 24;8(1):coaa056. doi: 10.1093/conphys/coaa056 (PMC7311831; doi:10.1093/conphys/coaa056)

Supplementary Material for Development and validation of protein biomarkers of health in grizzly bears

Table S1.1 Mean and standard deviation of body condition index (BCI), weight, and straight-line length of grizzly bears by season, sex, and age.

| Season | Sex | Age | BCI | Weight (kg) | Length (cm) |
| --- | --- | --- | --- | --- | --- |
| Hypophagia | Male | Adult | -0.04±0.98 | 187±64 | 175±13 |
|  |  | Sub-adult | -0.50±0.56 | 93±20 | 152±10 |
|  | Female | Adult | -0.37±0.49 | 100±19 | 155±7 |
|  |  | Sub-adult | -0.35±0.64 | 89±20 | 149±10 |
|  | Female with cubs | Adult | -0.37±0.68 | 101±19 | 156±9 |
| Early Hyperphagia | Male | Adult | 0.38±0.63 | 159±21 | 164±7 |
|  |  | Sub-adult | -0.57 | 94 | 153 |
|  | Female | Adult | N/A | N/A | N/A |
|  |  | Sub-adult | 0.09 | 79 | 140 |
|  | Female with cubs | Adult | N/A | N/A | N/A |
| Late Hyperphagia | Male | Adult | 1.05±1.28 | 225±33 | 177±6 |
|  |  | Sub-adult | 0.12±0.43 | 137±39 | 159±13 |
|  | Female | Adult | 0.94±0.62 | 137±17 | 159±5 |
|  |  | Sub-adult | 0.67±0.42 | 112±16 | 150±12 |
|  | Female with cubs | Adult | 1.46±0.69 | 147±23 | 158±7 |

Table S1.2 Final protein concentration (µg protein/mg tissue) of samples for each preparation method. In addition to traditional methods, two commercially available kit-based approaches were used: iST (Preomics, München, Germany) and S-Trap (Protifi, Huntington NY, USA). Two preparation methods 1) 1% Deoxycholate combined with the iST kit digestion and 2) the S-Trap did not yield accurate protein concentrations with the Bradford assay.

| **Method** | **Tissue Weight (mg)** | **Bradford Protein Concentration (µg/ul)** | **Total Protein Digested (µg)** | **Protein Concentration (µg protein/mg tissue)** |
| --- | --- | --- | --- | --- |
| 4.5M Urea | 16.00 | 0.75 | 89.91 | 179.82 |
| 9M Urea | 16.60 | 0.73 | 87.53 | 175.75 |
| 1% Deoxycholate | 16.70 | 0.14 | NA | 29.48 |
| 5% Sodium dodecyl sulfate | 20.00 | 0.77 | 92.29 | 184.58 |
| 5% Sodium dodecyl sulfate and iST kit | 20.00 | 1.75 | 90.00 | 140.36 |
| 10% Sodium dodecyl sulfate | 21.00 | 0.22 | NA | 53.80 |
| 10% Sodium dodecyl sulfate and iST kit | 21.00 | 0.89 | 90.00 | 71.08 |
| iST kit | 11.40 | 1.43 | 90.00 | 37.63 |

Table S1.3 Sample size corrected Akaike’s information criterion (AICc) for models within 2 AIC units of top model, Akaike weights (wi), number of parameters (K), log likelihood (LL) and parameters included in models for proteins related to energetics (n=6 proteins; n=111 individuals). The abbreviation BMA refers to bear management area (geographic area) and year and location on body refer to sample collection.

| Protein | Sex | Age Class | Sex*Age Class | Location on body | BMA | Season | Year | K | LL | AICc | Delta | w_i_ |
| --- | --- | --- | --- | --- | --- | --- | --- | --- | --- | --- | --- | --- |
| Adiponectin |  |  |  | x |  |  | x | 11 | -353.22 | 730.70 | 0.00 | 0.49 |
|  |  | x |  | x |  |  | x | 13 | -351.38 | 731.90 | 1.22 | 0.27 |
|  |  |  |  |  |  |  | x | 9 | -356.30 | 732.10 | 1.42 | 0.24 |
| Clusterin |  | x |  |  | x |  | x | 17 | -336.86 | 713.20 | 0.00 | 0.36 |
|  |  |  |  |  | x | x | x | 17 | -337.24 | 714.00 | 0.77 | 0.24 |
|  |  | x |  |  | x | x | x | 19 | -334.66 | 714.20 | 1.05 | 0.21 |
|  |  |  |  |  | x |  | x | 15 | -340.14 | 714.50 | 1.31 | 0.19 |
| Apolipoprotein B-100 |  | x |  |  | x |  | x | 17 | -213.66 | 466.80 | 0.00 | 0.39 |
|  |  | x |  | x | x |  | x | 19 | -211.29 | 467.50 | 0.71 | 0.27 |
|  | x | x |  |  | x |  | x | 19 | -211.71 | 468.30 | 1.54 | 0.18 |
|  | x |  |  | x |  | x |  | 9 | -224.52 | 468.50 | 1.74 | 0.16 |
| Alpha-1-acid glycoprotein |  | x |  | x |  |  |  | 7 | -501.71 | 1018.30 | 0.00 | 0.59 |
|  | x | x |  | x |  |  |  | 9 | -499.77 | 1019.00 | 0.69 | 0.41 |
| Transthyretin |  |  |  | x | x |  | x | 17 | -511.13 | 1061.70 | 0.00 | 1.00 |
| Vitamin D-binding protein |  |  |  | x |  | x | x | 13 | -507.46 | 1044.10 | 0.00 | 1.00 |

Table S1.4 Sample size corrected Akaike’s information criterion (AICc) for models within 2 AIC units of top model, Akaike weights (wi), number of parameters (K), log likelihood (LL) and parameters included in models for proteins related to reproduction (n=6 proteins; n=111 individuals). The abbreviation BMA refers to bear management area (geographic area) and year and location on body refer to sample collection.

| Protein | Sex | Age Class | Sex*Age Class | Location on body | BMA | Season | Year | K | LL | AICc | Delta | w_i_ |
| --- | --- | --- | --- | --- | --- | --- | --- | --- | --- | --- | --- | --- |
| Ceruloplasmin | x |  |  | x |  | x |  | 9 | -285.86 | 591.20 | 0.00 | 1.00 |
| Fetuin-B |  | x |  | x |  | x | x | 15 | -341.56 | 717.30 | 0.00 | 0.67 |
|  | x | x |  | x |  | x | x | 17 | -339.64 | 718.70 | 1.40 | 0.33 |
| Complement C3 |  | x |  | x |  | x | x | 15 | -415.38 | 865.00 | 0.00 | 1.00 |
| Afamin |  | x |  | x |  | x | x | 15 | -338.51 | 711.20 | 0.00 | 0.25 |
|  |  |  |  | x | x | x | x | 19 | -333.22 | 711.40 | 0.13 | 0.24 |
|  |  | x |  | x | x | x | x | 21 | -330.59 | 711.70 | 0.51 | 0.19 |
|  |  |  |  | x |  | x | x | 13 | -341.46 | 712.10 | 0.83 | 0.17 |
|  | x |  |  | x |  | x | x | 15 | -338.99 | 712.20 | 0.95 | 0.16 |
| Prostaglandin F synthase 1 |  |  | x |  |  |  |  | 8 | -95.95 | 209.10 | 0.00 | 0.21 |
|  | x |  | x |  |  |  |  | 8 | -95.95 | 209.10 | 0.00 | 0.21 |
|  |  | x | x |  |  |  |  | 8 | -95.95 | 209.10 | 0.00 | 0.21 |
|  | x | x | x |  |  |  |  | 8 | -95.95 | 209.10 | 0.00 | 0.21 |
|  |  | x | x |  |  | x |  | 10 | -94.56 | 211.00 | 1.87 | 0.08 |
|  | x | x | x |  |  | x |  | 10 | -94.56 | 211.00 | 1.87 | 0.08 |
| Serpin B5 (Maspin) |  |  |  |  |  | x |  | 5 | -440.19 | 890.90 | 0.00 | 0.36 |
|  | x |  |  |  |  | x |  | 7 | -438.05 | 891.00 | 0.17 | 0.33 |
|  |  | x |  |  |  | x |  | 7 | -438.70 | 892.30 | 1.46 | 0.17 |
|  |  |  |  |  |  |  |  | 3 | -443.31 | 892.80 | 1.96 | 0.14 |

Table S1.5 Sample size corrected Akaike’s information criterion (AICc) for models within 2 AIC units of top model, Akaike weights (wi), number of parameters (K), log likelihood (LL) and parameters included in models for proteins related to stress (n=6 proteins; n=111 individuals). The abbreviation BMA refers to bear management area (geographic area) and year and location on body refer to sample collection.

| Protein | Sex | Age Class | Sex*Age Class | Location on body | BMA | Season | Year | K | LL | AICc | Delta | w_i_ |
| --- | --- | --- | --- | --- | --- | --- | --- | --- | --- | --- | --- | --- |
| GRP78/BIP | x | x |  | x |  | x | x | 17 | -403.85 | 847.20 | 0.00 | 0.23 |
|  |  | x |  | x |  |  | x | 13 | -409.48 | 848.10 | 0.94 | 0.14 |
|  |  | x | x | x |  | x | x | 18 | -403.24 | 848.60 | 1.48 | 0.11 |
|  | x | x | x | x |  | x | x | 18 | -403.24 | 848.60 | 1.48 | 0.11 |
|  |  |  | x | x |  | x | x | 18 | -403.24 | 848.60 | 1.48 | 0.11 |
|  | x |  | x | x |  | x | x | 18 | -403.24 | 848.60 | 1.48 | 0.11 |
|  |  | x |  | x |  | x | x | 15 | -407.26 | 848.70 | 1.59 | 0.10 |
|  | x | x |  | x |  |  | x | 15 | -407.39 | 849.00 | 1.84 | 0.09 |
| α-2-macroglobulin |  | x |  | x |  | x |  | 9 | -374.32 | 768.10 | 0.00 | 1.00 |
| Annexin |  |  |  | x |  |  | x | 11 | -559.17 | 1142.60 | 0.00 | 0.34 |
|  | x |  |  | x |  | x | x | 15 | -554.58 | 1143.40 | 0.81 | 0.23 |
|  |  |  |  | x |  | x | x | 13 | -557.17 | 1143.50 | 0.92 | 0.22 |
|  | x |  |  | x |  |  | x | 13 | -557.20 | 1143.50 | 0.97 | 0.21 |
| CBG |  | x |  | x |  | x | x | 15 | -147.32 | 328.80 | 0.00 | 0.23 |
|  |  |  | x | x |  | x | x | 18 | -143.66 | 329.50 | 0.63 | 0.17 |
|  | x |  | x | x |  | x | x | 18 | -143.66 | 329.50 | 0.63 | 0.17 |
|  |  | x | x | x |  | x | x | 18 | -143.66 | 329.50 | 0.63 | 0.17 |
|  | x | x | x | x |  | x | x | 18 | -143.66 | 329.50 | 0.63 | 0.17 |
|  | x | x |  | x |  | x | x | 17 | -145.56 | 330.60 | 1.73 | 0.10 |
| Endoplasmin |  | x |  | x |  |  | x | 13 | -277.91 | 585.00 | 0.00 | 1.00 |
| Kininogen |  | x |  | x | x |  | x | 19 | -332.12 | 709.20 | 0.00 | 0.68 |
|  |  |  |  | x | x |  | x | 17 | -335.60 | 710.70 | 1.50 | 0.32 |
| SOD |  |  |  | x |  |  | x | 11 | -520.87 | 1066.00 | 0.00 | 1.00 |

Abbreviations are as follows: GRP78/BIP: 78 kDa glucose-regulated protein; CBG: corticosteroid-binding globulin; Endoplasmin: heat shock protein 90kDa beta member 1; SOD: Superoxide dismutase.

Figure S1.1 A single skin sample was either collected from the outside of the upper thigh (area above the knee on the hind leg) and/or from the external ear surface (boxed in red) of a grizzly bear using a 4-6 mm biopsy punch.


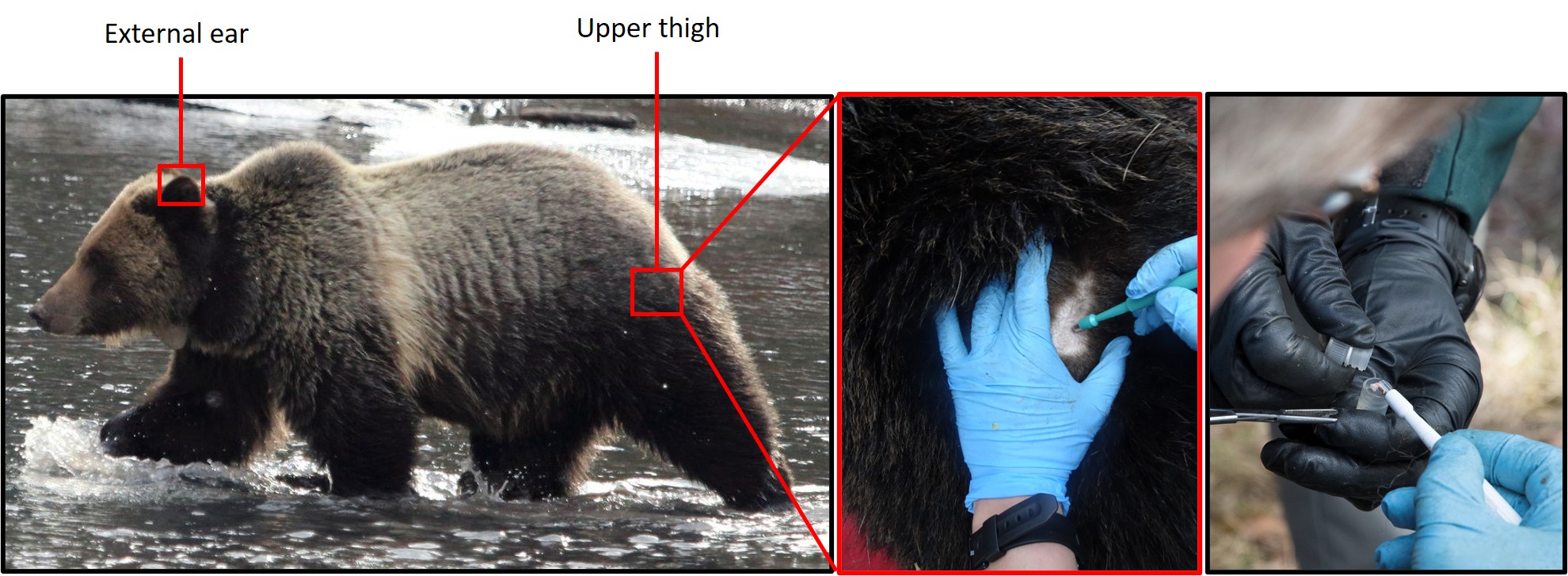


Figure S1.2 Workflow of sample optimization methods. In addition to traditional methods, two kit-based approaches were used: iST (Preomics, München, Germany) and S-Trap (Protifi, Huntington NY, USA).


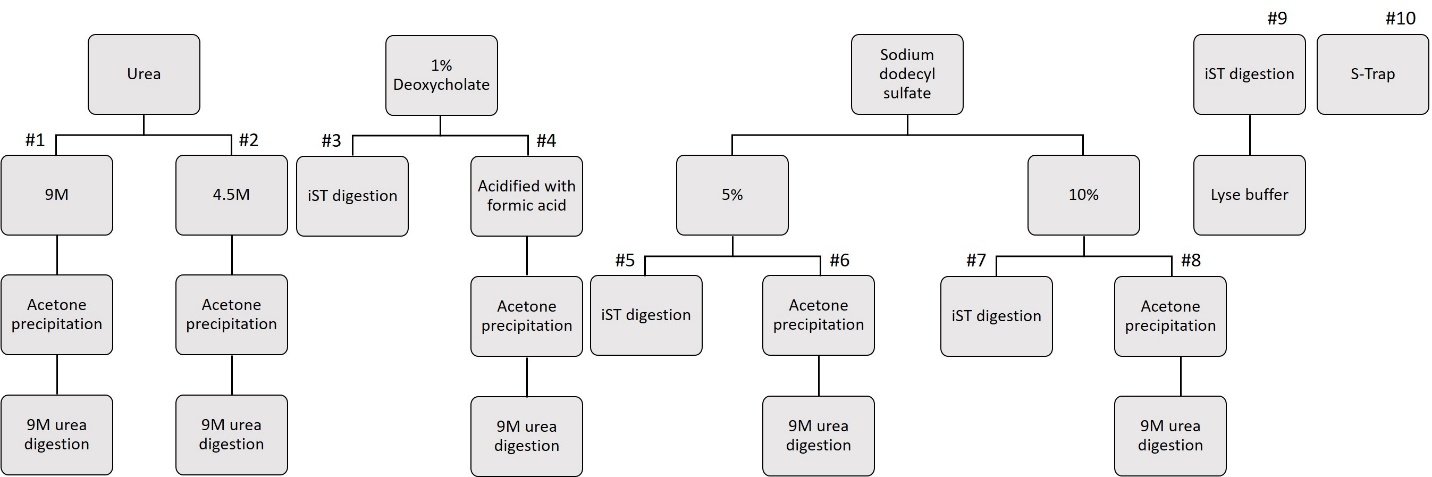


Figure S1.3 Slope graphs for individual bears that show the relationship between repeated samples across years. All skin samples were collected from the outer thigh.


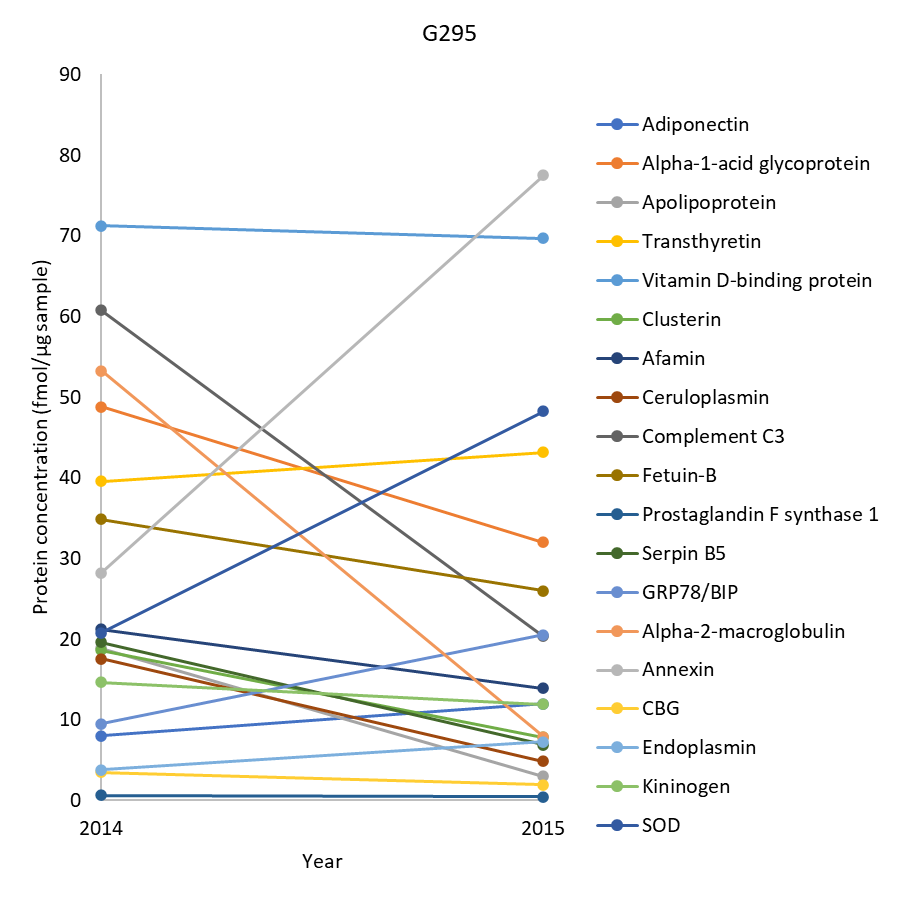


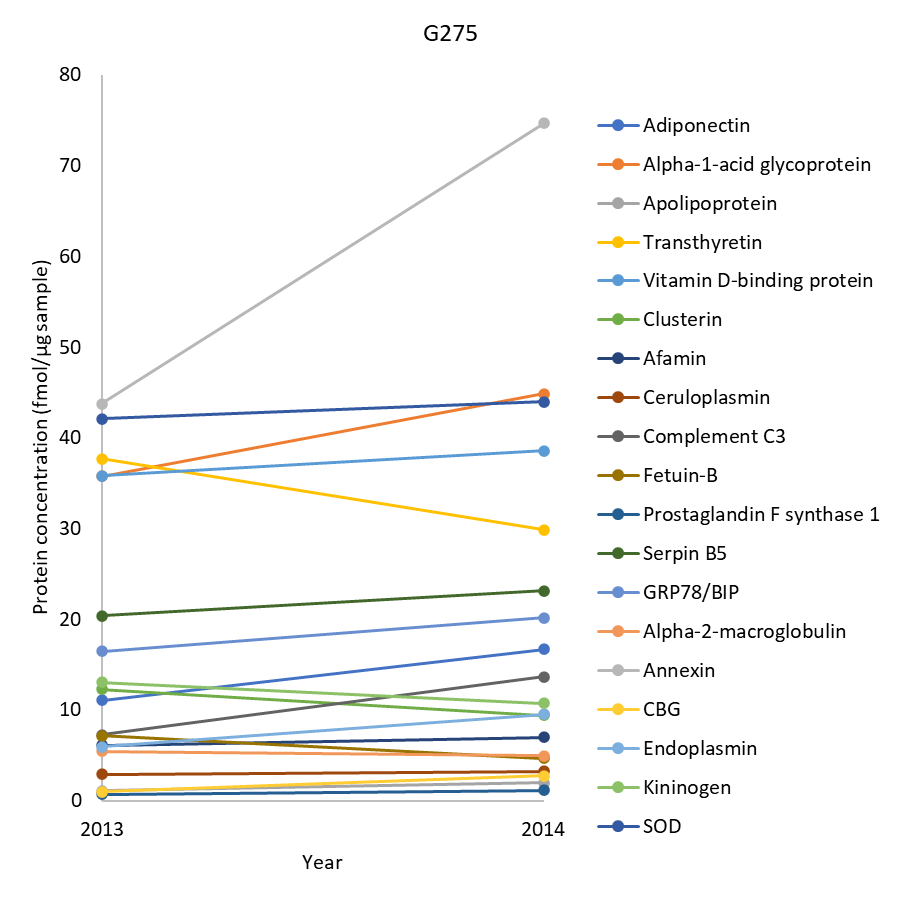


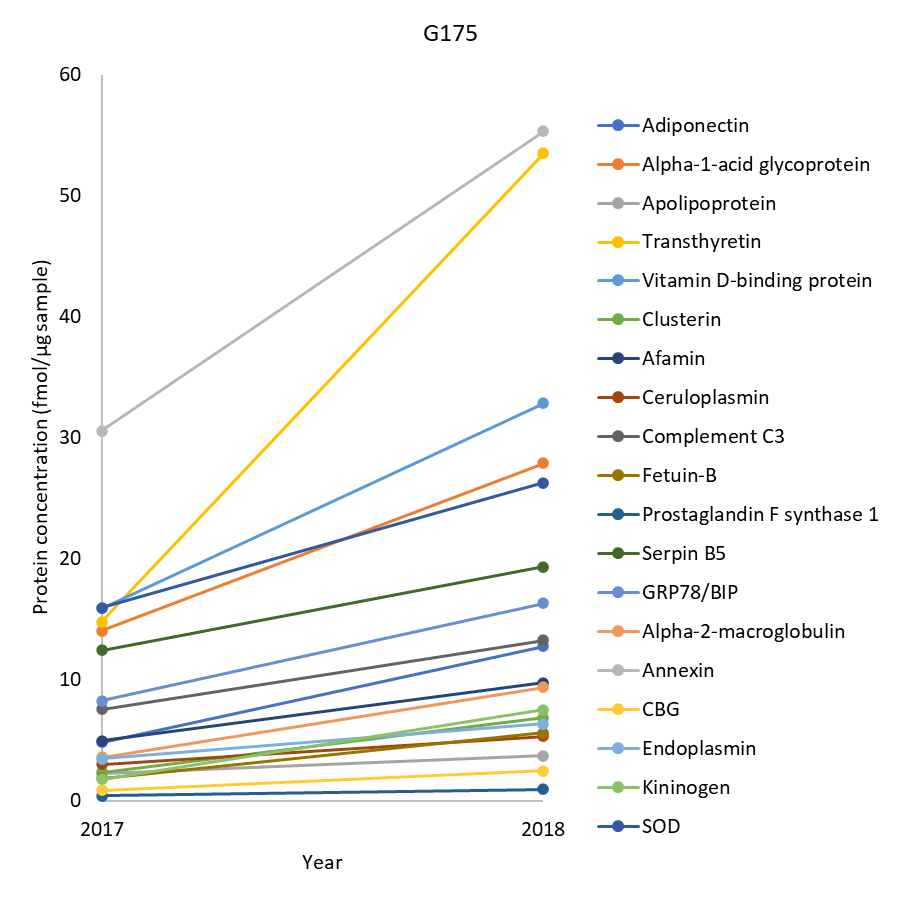


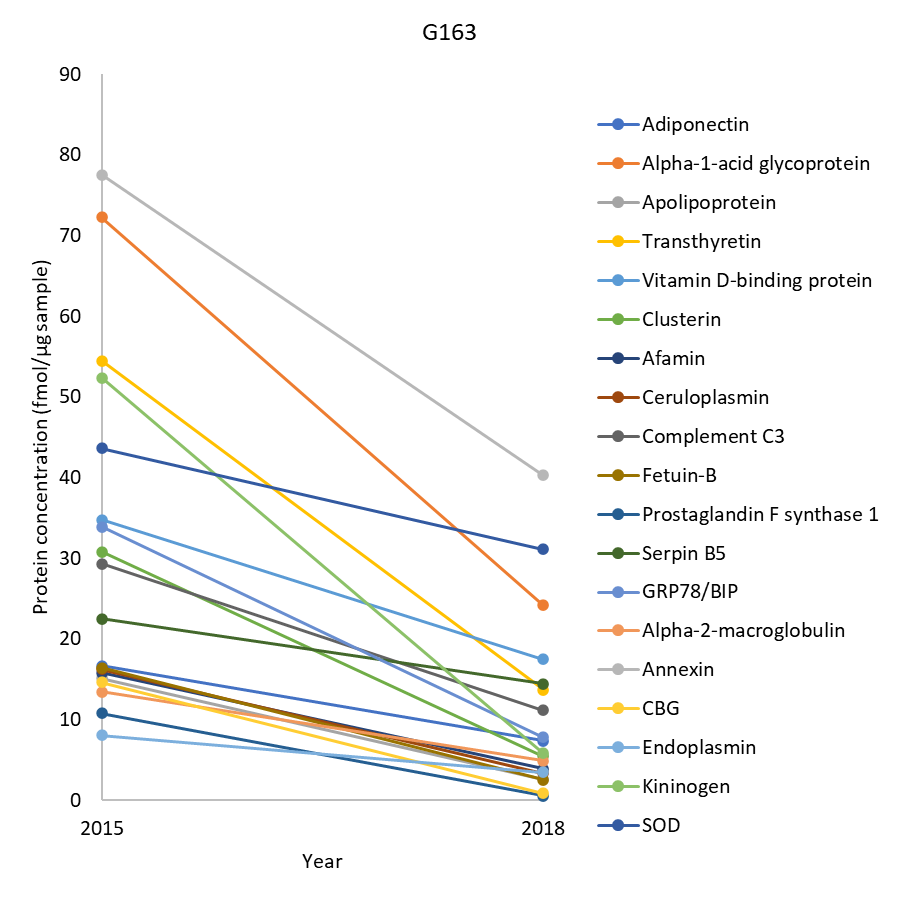


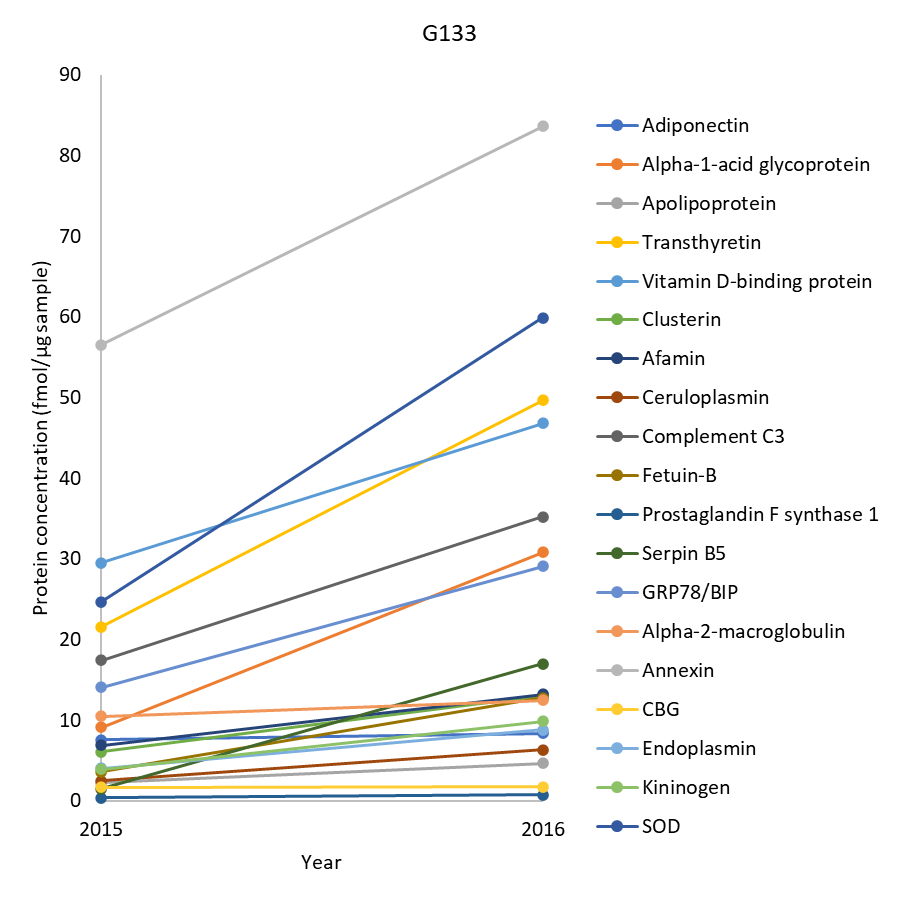


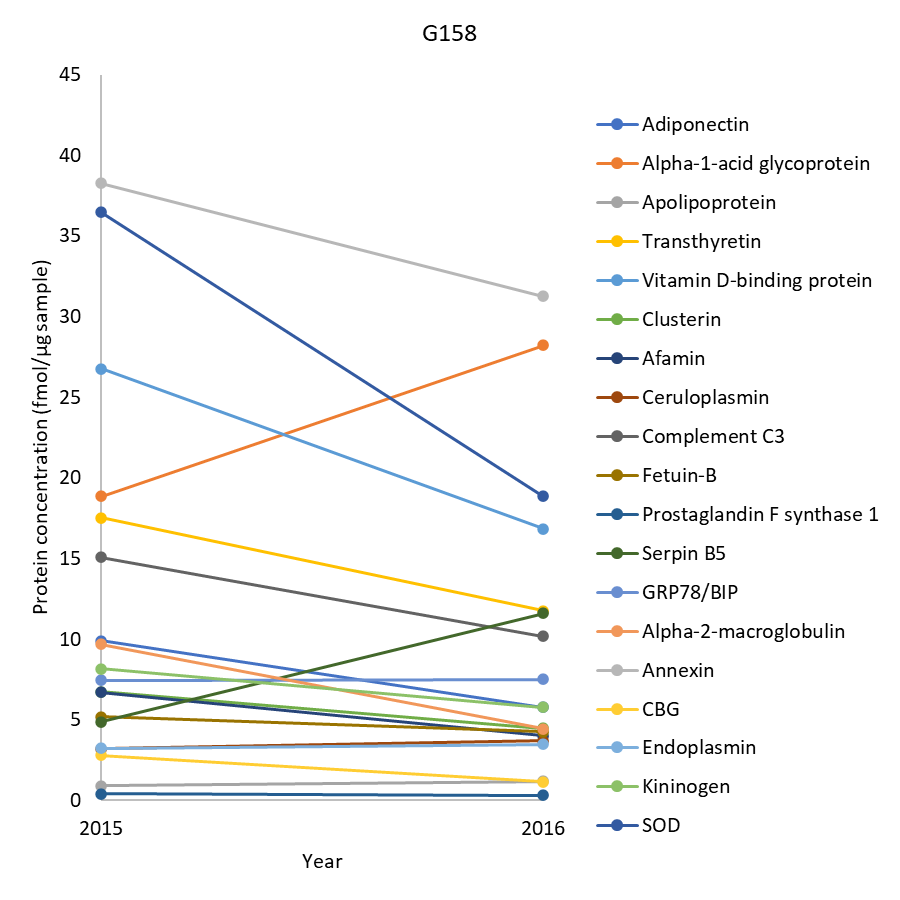


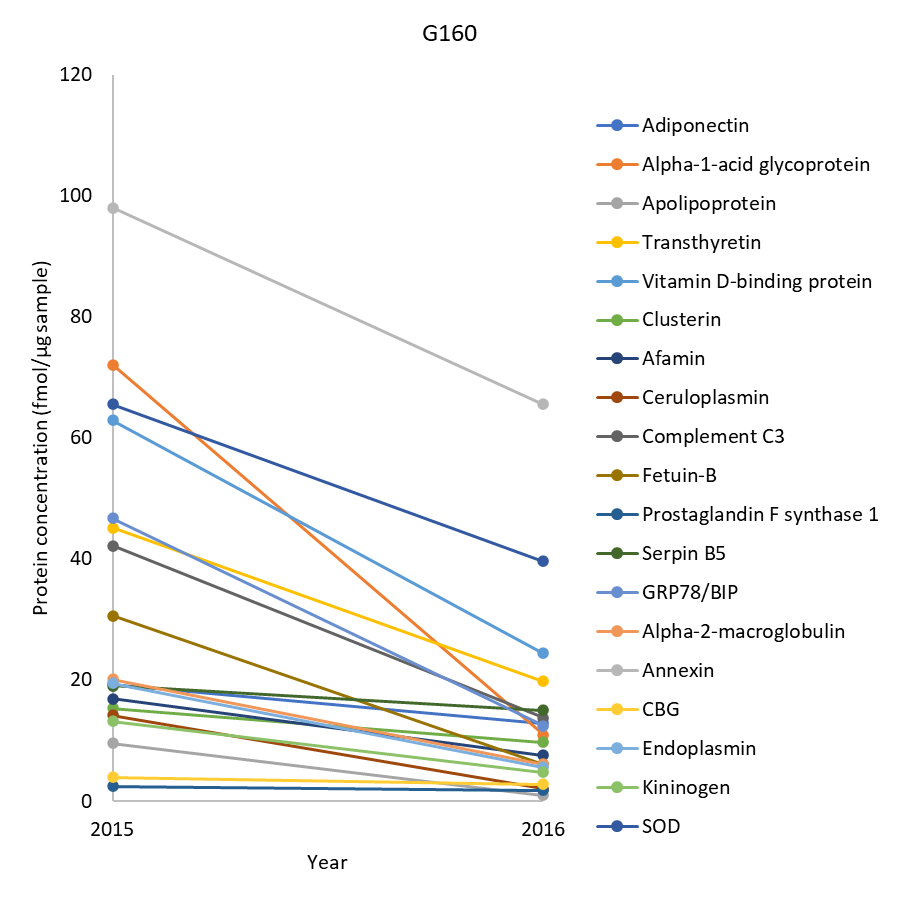


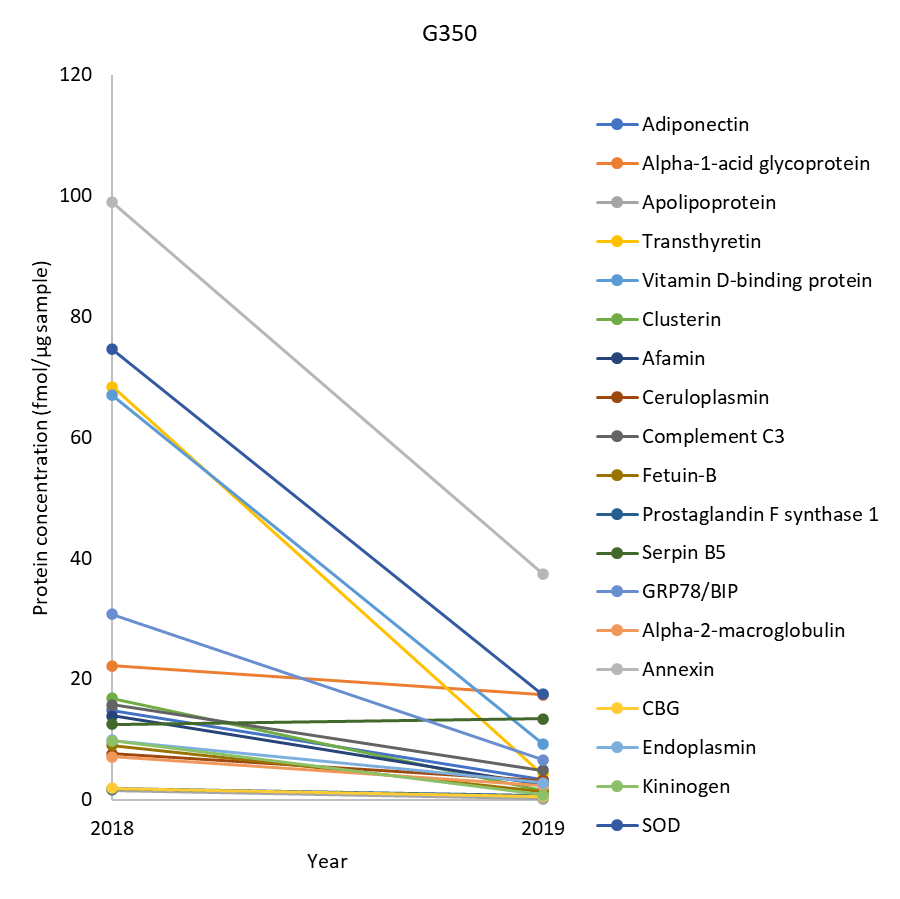

Supplement: CONPHYS-2019-167_R2_Supplementary [file conphys-2019-167_r2_supplementary.docx]
